# Donor‐π‐Acceptor Photoinitiators for High‐Efficiency Visible LED and Sunlight Polymerization and High‐Precision 3D Printing

**DOI:** 10.1002/anie.202425198

**Published:** 2025-05-02

**Authors:** Ji Feng, Tong Gao, Fabrice Morlet‐Savary, Michael Schmitt, Celine Dietlin, Jing Zhang, Xiaotong Peng, Pu Xiao, Frédéric Dumur, Jacques Lalevée

**Affiliations:** ^1^ Université de Haute‐Alsace, CNRS IS2M UMR7361 Mulhouse F‐68100 France; ^2^ Université de Strasbourg France; ^3^ Future Industries Institute University of South Australia Mawson Lakes SA 5095 Australia; ^4^ State Key Laboratory of High‐Performance Ceramics and Superfine Microstructure, Shanghai Institute of Ceramics Chinese Academy of Sciences Shanghai 200050 P.R. China; ^5^ Aix Marseille Univ, CNRS, ICR UMR 7273 Marseille F‐13397 France

**Keywords:** 3D Printing, LED, Photoinitiator, Photopolymerization, Sunlight

## Abstract

This study presents the development and evaluation of five dyes with varying conjugated energy levels and donor‐π‐acceptor (D‐π‐A) structures as photoinitiators for free radical polymerization. Their photoinitiation efficiencies are systematically assessed under both visible‐light LED and sunlight. Notably, the conversions reach up to 81% within just 30 s under sunlight, demonstrating the ultrafast and efficient polymerization capabilities of the dyes. The efficient electron transfer is facilitated by the D‐π‐A structure, where the conjugation is reduced or interrupted by the high distortion between the electron‐withdrawing and the electron‐releasing units. This distortion can prevent the overlap of frontier molecular orbitals, decreasing the energy difference between the ground state and the excited state of dyes, thereby enhancing the electron transfer reactivity with additives. Additionally, we propose a chemical mechanism for the electron transfer reaction in the three‐component systems. The study also explores the application of naphtho[2,3‐*d*]thiazole‐4,9‐dione‐based dyes as donors in additive manufacturing demonstrating their effectiveness in three different 3D printing technologies, i.e., direct laser writing (DLW), digital light processing (DLP), and liquid crystal display (LCD). These three‐component formulations achieve high‐precision 3D printed objects, with detailed characterization and comparison of the resulting structures.

## Graphical abstract

The proposed D‐π‐A structured dyes enable efficient electron transfer with additives, thanks to their tailored architecture, making them ideal for high‐performance photoinitiating systems. They allow the fabrication of high‐precision 3D printed objects and exhibit outstanding performance under sunlight.

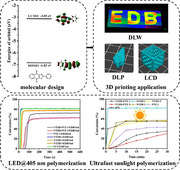

## Introduction

Since the 21st century, photopolymerization has garnered a significant attention from both academia and industry, leading to its widespread application, particularly in the fields of biomedical materials, nanotechnology, and 3D printing^[^ ^1^, ^2^, ^3^ ^]^ The efficacy of photopolymerization mainly depends on the light source and photoinitiators (PIs). Traditionally, mercury lamps, operating primarily in the ultraviolet (UV) range, have been the main light source for photopolymerization; however, their high cost and environmental impact have prompted interest in safer, more sustainable alternatives^[^ ^4^, ^5^ ^]^ Visible‐light LEDs have emerged as a promising option due to their low costs, environmental friendliness and reduced phototoxicity compared to the traditional high‐energy mercury lamps. These LEDs can be operated for extended periods without adverse health or environment effects, making them a promising option for various applications.^[^ ^6^ ^]^ However, the emission wavelength of visible‐light LEDs, generally above 400 nm, poses a compatibility issue with conventional PIs, which are optimized for UV sources. As a result, most commercially available PIs cannot be utilized for polymerization with visible‐light LEDs.^[^ ^7^ ^]^ This has created an urgent need to develop novel PIs or photoinitiating systems (PISs) that are specifically tailored for visible‐light LED photopolymerization.

To address this need, the design of effective PIs and PISs focuses on achieving a separation between the highest occupied molecular orbital (HOMO) and the lowest unoccupied molecular orbital (LUMO) to minimize the overlap of the frontier molecular orbitals.^[^ ^8^ ^]^ This can be achieved by designing D‐π‐A structures that absorb wavelengths in the visible range, facilitated by introducing spatial site resistance and distortions between donors and acceptors. This design helps create small energy barriers, making the PIs easier to excite. Alternatively, effective PISs can also be achieved using coinitiators (e.g., ethyl 4‐dimethylaminobenzoate (EDB) and *bis*(4‐*tert*‐butylphenyl)iodonium hexafluorophosphate (Iod)), which facilitate electron transfer. Despite these innovations, achieving effective visible LEDs photopolymerization remains challenging.

In addition to visible‐light LEDs, sunlight presents a renewable and cost‐effective light source for photopolymerization, offering substantial potential for sustainable photopolymerization techniques.^[^ ^9^, ^10^ ^]^ However, the limited availability of PIs and PISs designed for sunlight has hindered their broader applications.^[^ ^11^ ^]^ Although Type I PIs can undergo a direct bond‐homolytic cleavage without additives to generate free radicals to initiate photopolymerization,^[^ ^7^ ^]^ their reactivity under sunlight is limited. By contrast, Type II PIs, are more suitable for sunlight‐induced polymerization as they can be stabilized under sunlight without the addition of additives. However, most reported PI dyes that can initiate polymerization under sunlight often exhibit a low reactivity and require a prolonged sunlight exposure to be effective.^[^ ^12^ ^]^ Thus, developing stable and environmentally friendly systems for efficient sunlight‐induced polymerization remains a priority, with photocatalysts offering promising storage stability under both sunlight and air.

3D printing, a transformative application of photopolymerization technology, has become a focal area in advanced manufacturing.^[^ ^13^, ^14^ ^]^ Typically, 3D models are designed using computer modeling software. The model is then sliced into thin layers, enabling it to be printed layer by layer.^[^ ^15^, ^16^ ^]^ Among 3D printing techniques, light‐curing 3D printing stands out for its speed, precision, and ability to create intricate microstructures, positioning it as a powerful tool in fields such as aerospace and biomedicine,^[^ ^17^, ^18^, ^19^, ^20^ ^]^ This manufacturing method is based on photopolymerization techniques in which photosensitive resins are activated and cured by light irradiation. Depending on the different principles of pattern illustration and control systems, light‐cured 3D printing can be categorized into a number of technologies, for instance, DLW, DLP and LCD. and other printing technologies.^[^ ^15^ ^]^ However, limitations of current PISs can lead to incomplete resin curing and compromised print quality. Meanwhile, few PISs are compatible with multiple printing technologies simultaneously. Therefore, it is essential to develop an efficient PISs that can be applied across various light‐curing 3D printing techniques (e.g., LCD, DLP, and DLW).

**Scheme 1:**
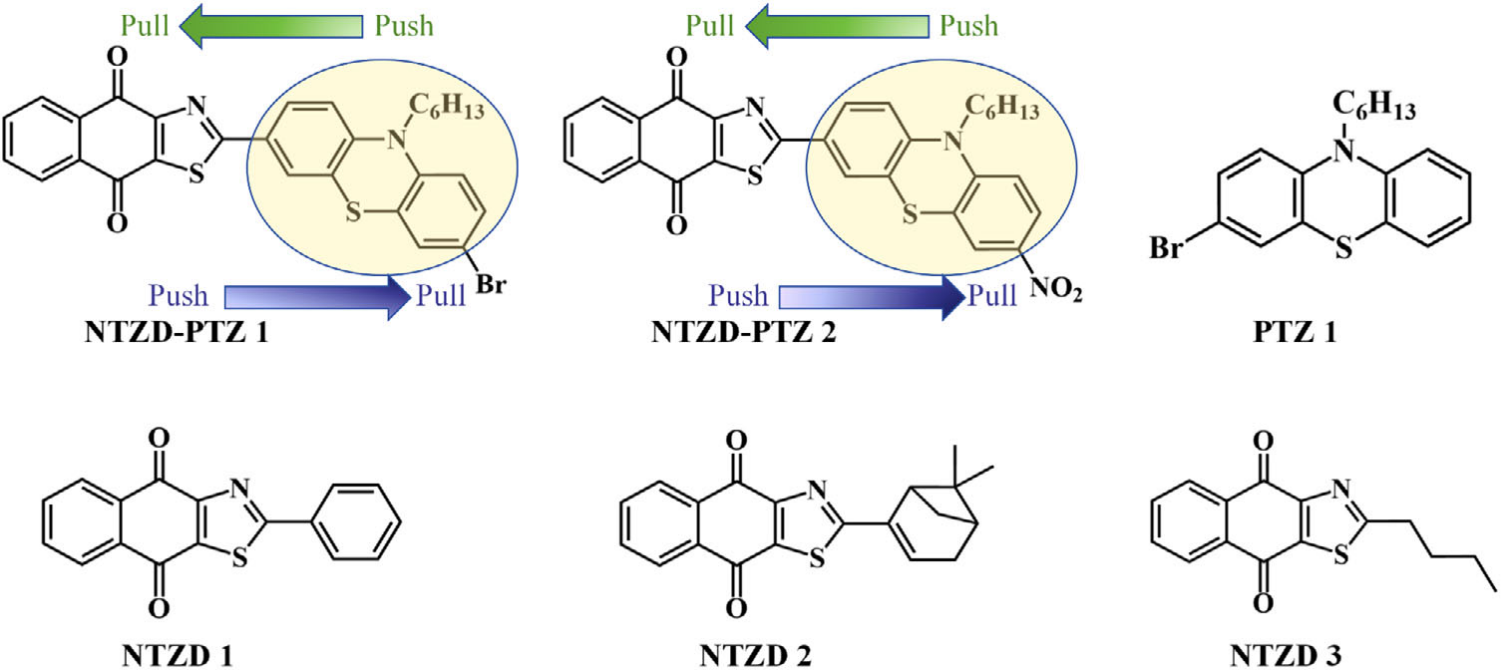
Chemical structures of dyes.

Herein, we propose a new strategy for designing D‐π‐A organic dyes by combining various donor and acceptor groups, as illustrated in Scheme 1. Specifically, five new dyes with D‐π‐A structures are successfully designed and easily synthesized using **n**aphtho[2,3‐***d***]**t**hia**z**ole‐4,9‐**d**iones (NTZD) as acceptor (A) groups. These dyes formed various conjugated structures with the donor groups phenothiazine (NTZD‐PTZ 1 and 2), phenyl (NTZD 1), *n*‐butyl (NTZD 3), and the product using R myrtenal (NTZD 2). For comparison, the phenothiazine derivative (PTZ 1) was also examined as the sixth dye. In addition, the D‐π‐A structures can be conveniently tailored to absorb wavelengths in the visible region, such as 405 nm or above.^[^ ^21^ ^]^ More importantly, this conjugation‐breaking reduces the overlap between HOMO and LUMO due to the steric hindrance between the donors and the acceptors, thus facilitating the achievement of a small ΔE_S‐T_ and making electron transfer easier to occur.^[^ ^22^, ^23^, ^24^ ^]^ Meanwhile, a new chemical mechanism for electron transfer reactions in three‐component systems is proposed. High functional conversions (FCs) of trimethylolpropane triacrylate (TMPTA) C═C double bonds obtained by photopolymerization of six dyes as PIs under visible 405 nm LED and sunlight confirm their excellent photoinitiation capacity. Furthermore, successful application of NTZD 1 in 3D printing yielded high‐resolution patterns compatible across multiple light‐curing techniques, including DLW, DLP, and LCD screen. This work suggests that D‐π‐A structures tailored for specific absorption wavelengths hold considerable promise for broader applications in photopolymerization and beyond.

## Experimental Process

### Dyes and Other Materials

A collection of six dyes used for photopolymerization (under UV–vis light and under sunlight in the air) were successfully synthesized. The synthetic routes used to prepare these compounds are detailed in Section 3.1 and Supporting Information below. Other materials, the monomer, i.e., TMPTA was obtained from Sartomer (France). The storage inhibitor was not removed. The benchmark commercial photoinitiator 2‐isopropylthioxanthone (ITX) used for comparison was obtained from Sartomer–Lambson (United Kingdom). Ethyl 4‐dimethylaminobenzoate (EDB) used as the electron donor and *bis*(4‐*tert*‐butylphenyl)iodonium hexafluorophosphate (Iod) as the electron acceptor were purchased from Sartomer–Lambson (United Kingdom).

### The UV and Fluorescence Properties of Dyes

The UV–vis absorption properties and UV steady‐state photolysis of the six dyes, dissolved in dichloromethane (DCM) at a concentration of 2.5 × 10^−5^ M, were studied using a JASCO V730 spectrophotometer. Steady‐state photolysis of the different systems (dye, dye/EDB, dye/Iod) were investigated upon irradiation (e.g., under LED@405 nm), the concentration of the additives was 5 × 10^−5^ M. The fluorescence spectra of the different dyes were measured using the JASCO FP‐6200 fluorescence spectrophotometer, and the fluorescence excited state lifetimes were determined using the HORIBA PPD‐850 fluorimeter (The concentration of dye was 2.5 × 10^−5^ M in dichloromethane). Fluorescence quenching experiments were performed using the JASCO FP‐6200 spectrofluorometer, allowing for the extraction of pertinent parameters such as the electron transfer quantum yields (ϕ_et_) using the Equation 1. The Stern–Volmer coefficients (*K*_sv_) correspond to the slopes of the Stern–Volmer treatment in the fluorescence quenching experiments.

(1) $$\;\phi_{et}=\frac{K_{sv}\left[\mathrm{additive}\right]}{1+K_{sv}\left[\mathrm{additive}\right]}$$

The fluorescence quantum yield of the dye was determined using the one‐point method.^[^ ^25^ ^]^ Anthracene was chosen as the reference fluorescent standard, which according to the literature was reported to have a fluorescence quantum yield of 0.27.^[^ ^26^, ^27^ ^]^ Specifically, the integrated fluorescence intensity (i.e., the area encompassed by the fluorescence spectra) of the dye and the anthracene solution, respectively, as well as the absorbance of the incident light to one and the same excitation wavelength (350 nm) were measured, and the fluorescence quantum yield of the dye could be obtained by bringing in the following Equation 2.^[^ ^25^ ^]^ In particular, the UV absorbance of the samples at the excitation wavelength was kept around 0.1 in the experiment. The smaller absorption value avoids the internal filter effect, i.e., reabsorption of the emitted fluorescence, and more importantly ensures a linear scale between the absorption value and the fluorescence signal.^[^ ^28^ ^]^

(2) $$Q=Q_{R}\;\frac{I}{I_{R}}\frac{OD_{R}}{OD}\frac{n^{2}}{n_{R}^{2}}$$

where *Q* is the fluorescence quantum yield, *I* is the fluorescence spectral area, *n* is the refractive index of the solvent, and OD is the UV–vis absorption intensity. The subscript R refers to anthracene. The solvent used was ethanol.

### Redox Potentials of Dyes Obtained by Cyclic Voltammetry (CV)

Redox potentials (oxidation is *E*_ox_, reduction is *E*_red_) of dyes were measured by CV. The different dyes and tetrabutylammonium hexafluorophosphate used as the support electrolyte were dissolved in acetonitrile under nitrogen atmosphere. The singlet excited state energy (*E*_S1_) of the dyes was determined from the intersection of the normalized UV–vis absorption and fluorescence spectra. Similarly, the triplet energy (*E*_T1_) determined by molecular modeling (Gaussian 03). According to the Rehm–Weller equation,^[^ ^29^ ^]^ the change of free energy from the singlet excited state (Δ*G* ^S1^_EDB_ or Δ*G* ^S1^_Iod_) and the change of free energy from the triplet excited state (Δ*G* ^T1^_EDB_ or Δ*G* ^T1^_Iod_) in the electron transfer reaction between dyes and additives can be calculated. In particular, the *E*_ox_ of amine (EDB) is 1.0 V, and *E*_red_ of iodonium salt (Iod) is −0.7 V.^[^ ^30^ ^]^

### Electron Spin Resonance Spin Trapping (ESR‐ST) Experiments

To detect the radicals formed by reaction between dye and additives (EDB or Iod), ESR‐ST were carried out using an X‐band spectrometer (Bruker EMX‐plus). Precisely, the solutions were prepared with the dye/EDB and dye/Iod systems with concentrations of 2 × 10^−4^ M in *tert*‐butylbenzene, and the spin trap agent phenyl‐*N‐tert*‐butylnitrone (PBN) was added to them, and the concentration of PBN was about 5 × 10^−4^ M. Then, upon irradiation at 405 nm and under nitrogen, the generation of free radicals could be observed.

### Visible‐Light LED and Sunlight Polymerization

In this study, one‐component (dye‐alone), two‐component (dye/EDB and dye/Iod) and three‐component system (dye/EDB/Iod) were prepared respectively, the monomers used in photopolymerization was TMPTA. Specifically, dye and additives (EDB/Iod) were dissolved in TMPTA and placed in a sealed vial, then stirred overnight in the dark. Here, the weight contents of dye, EDB, and Iod were calculated based on the weight of monomers, and the weight content of dyes was set to 0.01 wt%, and the weight content of additive was set to 1 wt%. Notably, the formulations should be protected from light all the time before use. Then, the photopolymerization capacity of the prepared formulations under the irradiation of visible‐light LED (405, 450 nm) was tested (*I*_405 nm_ = 110 mW cm^−2^; *I*_450nm_ = 50 mW cm^−2^). Specifically, five drops of the prepared formulations were dropped into a self‐made plastic mold (a circular mold with a depth of 2 mm and a diameter of 10 mm), and the characteristic peak of acrylate functional group was continuously detected at about 6150 cm^−1^ by Real‐Time Fourier Transform Infrared spectroscopy (RT‐FTIR, JASCO FTIR‐6000). The conversion of monomer functional groups is calculated by the following equation:

(3) $$\mathrm{Conversion}\;\left(t\right)=\left(1-\frac{A\left(t\right)}{A0}\right)\;\times 100\%$$

where A_0_ is the initial peak area before light irradiation and A(t) is the peak area after being irradiated with light for t s.

In addition to the polymerization experiment under artificial light generated by an LED, we also explored the sunlight‐induced photopolymerization with the same three‐component formulation as mentioned above. The polymerization of samples with different thicknesses (2 and 4 mm respectively) was tested. RT‐FTIR spectra were also used to monitor the conversion of acrylate functional groups. Sunlight‐induced polymerization was carried out in Mulhouse (+77 43 ′ E, 47 75 ′ N) in eastern France on August 30th, 2024. The weather conditions were sunny that day, and the temperature was around 20 °C. As a result of these weather conditions, the irradiance was in the range of 800 W m^−2^.^[^ ^31^ ^]^

### Application Comparison of Different 3D Printing Techniques

To print 3D objects with high resolution and precision, we optimized the parameters of 3D printing, and the best parameters obtained are described in detail below. In this study, three 3D printing techniques were compared: single‐layer 3D printing via DLW using a NEJE DK‐8‐KZ device, DLP with a projector‐based system (Anycubic Photon D2, China), and LCD 3D printing (Anycubic Photon Mono, China). Photosensitive formulations were optimized for each technique. For DLW, two formulations were developed: NTZD‐PTZ 1/EDB/Iod (0.05 wt%/1 wt%/1 wt%) and NTZD‐PTZ 2/EDB/Iod (0.05 wt%/1 wt%/1 wt%), both dissolved in trimethylolpropane triacrylate (TMPTA). These mixtures were poured into a square tank (3 × 2 × 0.3 cm). Printing was conducted using NEJE software with the following parameters: X and Y tables controlled by a computer‐operated 405 nm laser diode, a burning time of 20 s (assumed units), and a design of 3D numbers (“938”) and letters (“EDB”) with a uniform thickness of 0.3 cm. The total printing time was approximately 5 min. For DLP, the formulation consisted of NTZD‐PTZ 1/EDB/Iod (0.05 wt%/1 wt%/1 wt%) dissolved in TMPTA. This mixture was poured into the resin tank of a DLP 3D printer (Anycubic Photon D2, China). Printing parameters were set as follows: a curing time of 70 s per layer, a layer thickness of 20 µm, five bottom layers with a curing time of 100 s, a light‐off time of 0.5 s, and a total printing duration of 6 h and 26 min. The resulting model measured 20 × 10 × 6 mm. For LCD, the formulation same to that of DLP – NTZD‐PTZ 1/EDB/Iod (0.05 wt%/1 wt%/1 wt%) in TMPTA – and was poured into the resin tank of a LCD 3D printer (Anycubic Photon Mono, China). Printing parameters included a curing time of 30 s per layer, a layer thickness of 100 µm, three bottom layers with a curing time of 200 s, a light‐off time of 2 s, and a total printing time of 1 h and 50 min. The resulting model had dimensions of 15 × 15 × 15 mm.

The 3D patterns printed by DLW technique were characterized by digital optical microscope (DSX‐HRSU of Olympus). Surface accuracy and resolution of 3D objects printed by DLP and LCD techniques were characterized by scanning electron microscope (SEM).

### Computational Procedure

Gaussian 16 was used to optimize the geometry of dyes at the B3LYP/6–31G* level of theory to determine the triplet energies. Orbitales have then been computed in single point at a MPW1PW91/6–31G* level of theory.

## Results and Discussion

### Molecular Design and Theoretical Calculations

**Scheme 2:**
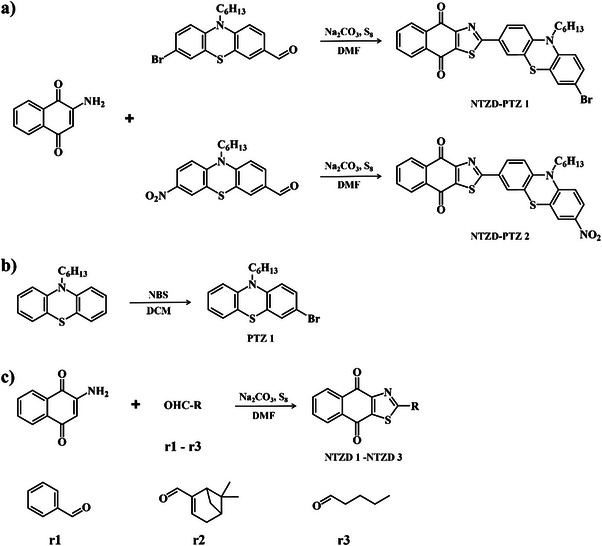
a) Synthetic routes to NTZD‐PTZ 1 and NTZD‐PTZ 2; b) synthetic routes to PTZ 1; and c) synthetic routes to NTZD 1, 2, and 3.

Scheme 2 outlines the synthetic routes of all dyes. First, in Scheme 2a, both NTZD‐PTZ 1 and NTZD‐PTZ 2 were prepared using phenothiazine as the electron donor group and naphtho[2,3‐*d*]thiazole‐4,9‐dione group as the electron acceptor group to construct D‐π‐A structure. Phenyl as aromatic (NTZD 1), butyl as aliphatic (NTZD 3) and the product with myrtenal (biobased) as conjugated double bond (NTSD 2) were also successfully prepared, shown in Scheme 1. PTZ 1 was used as a phenothiazine reference compound. In Scheme 2b, the synthetic route of PTZ 1 was directly synthesized from 10‐hexyl‐10*H*‐phenothiazine and *N*‐bromosuccinimide (NBS) in one step. NTZD 1–3 were prepared in one step starting from 2‐amino‐1,4‐naphthoquinone and the appropriate aldehyde r1–r3 by using the previously reported synthetic route^[^ ^32^ ^]^ (See Scheme 2c). It is worth noting that NTZD 1–3 also exhibits a D‐π‐A structure, which is different from NTZD‐PTZ 1 and NTZD‐PTZ 2 in that it lacks a larger conjugated structure.

**Figure 1:**
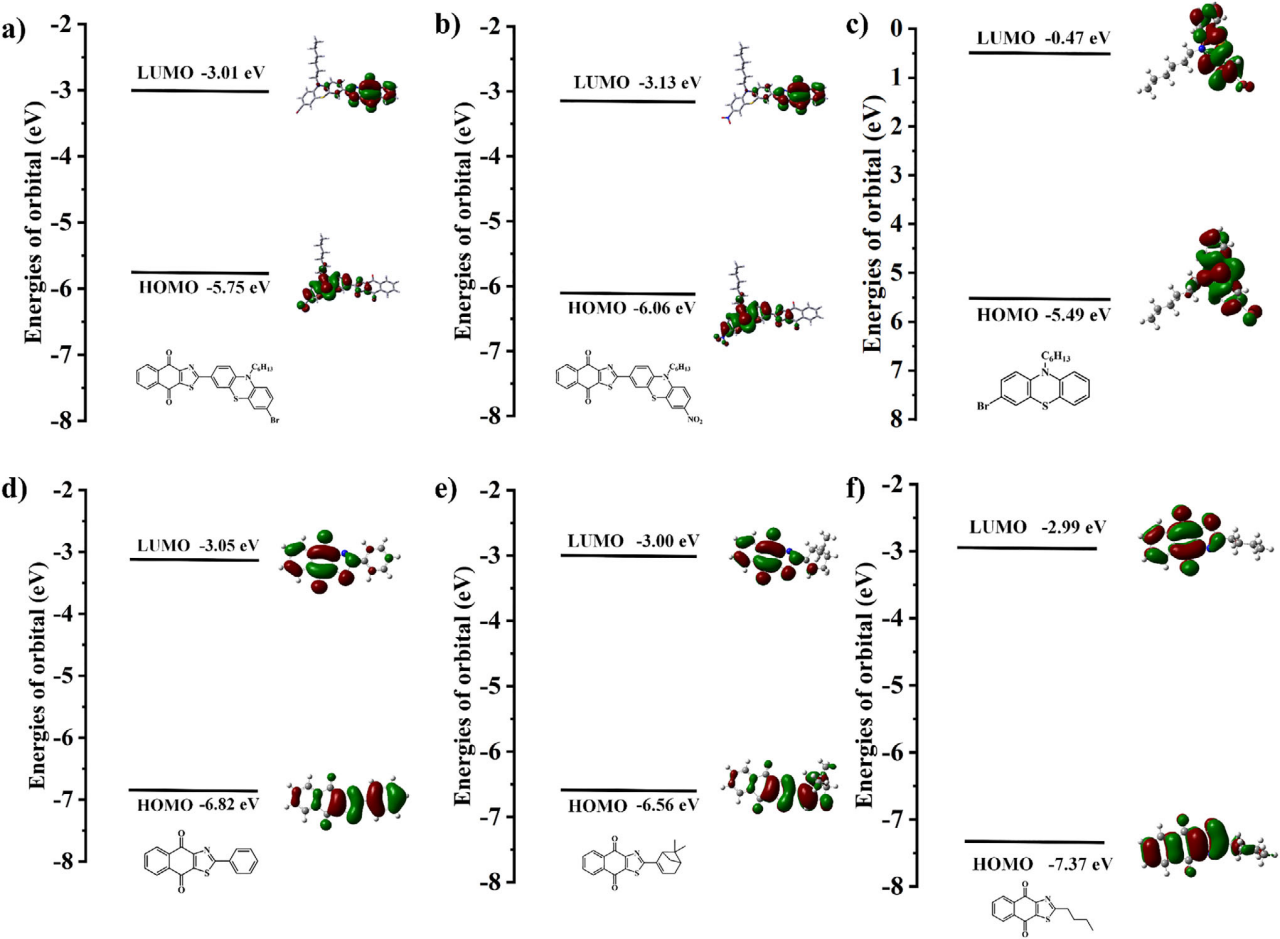
Frontier molecular orbital diagrams of a) NTZD‐PTZ 1, b) NTZD‐PTZ 2, c) PTZ 1, d) NTZD 1, e) NTZD 2, and f) NTZD 3.

To gain insights into the electron transitions related to the absorption bands of the six dyes designed, we performed computational simulations to optimize the geometric structure and to obtain the excited singlets. Figure 1 illustrates the distribution of electron clouds in frontier orbitales and the energy difference between the singlet and the triplet excited state. From the HOMO and the LUMO, it can be seen that the HOMO is located on the phenothiazine and other aryl‐ring or carbon‐chain part, while the LUMO is located on the naphtho[2,3‐*d*]thiazole‐4,9‐diones part. Except for PTZ 1, as expected in the design, the complete isolation of electron release and electron extraction was observed through a highly twisted structure and a large conjugated system. Moreover, it was well‐marked that the clear charge transfer characteristics correspond to the electron delocalization at the photoinduced HOMO‐LUMO transition (π→π* lowest energy transition).^[^ ^22^, ^33^ ^]^ According to the calculation in Figure 1, the order of the energy gap (*E*_g_) between HOMO and LUMO was consistent with the observation trend of its maximum absorption wavelength. The smaller the *E*_g_ between HOMO and LUMO, the longer the maximum absorption wavelength is expected to be.^[^ ^34^ ^]^ From Table 1, it can be seen that the color of the dissolved dye gradually changed from colorless to yellow and then to red, which was highly consistent with the calculation results. Here, because there was no large conjugated structure for electron transition in the PTZ 1 structure, its maximum absorption wavelength was the shortest. Molecular simulation also illustrated that the LUMO energy level of NTZD‐PTZ 2 was lower than the one of NTZD‐PTZ 1 (−3.13 eV vs. −3.01 eV), which can be attributed to the stronger electron‐withdrawing ability of nitro group compared with bromine. Under the same conditions, the addition of bromine groups also led to a slight red shift in the spectrum (494 nm for NTZD‐PTZ 1 and 472 nm for NTZD‐PTZ 2).^[^ ^22^ ^]^ These findings highlighted the good consistency between our calculated results and the experimental results. To sum up, in view of their absorption properties in the visible region, it can be inferred that these dyes had promising potential to initiate photopolymerization under ultraviolet and visible‐light or even sunlight irradiation.

**Table 1:** Correlation between energy difference between HOMO and LUMO and maximum absorption wavelength.

	NTZD‐PTZ 1	NTZD‐PTZ 2	NTZD 2	NTZD 1	NTZD 3	PTZ 1
	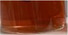	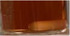	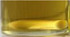	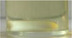	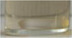	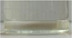
**Δ*E*_HOMO‐LUMO_ (eV)**	2.74	2.93	3.56	3.77	4.38	5.02
***λ*_max_ (nm)**	494	472	440	400	335	320

### Photophysical Properties of Dyes (Light Absorption and Emission Properties)

**Figure 2:**
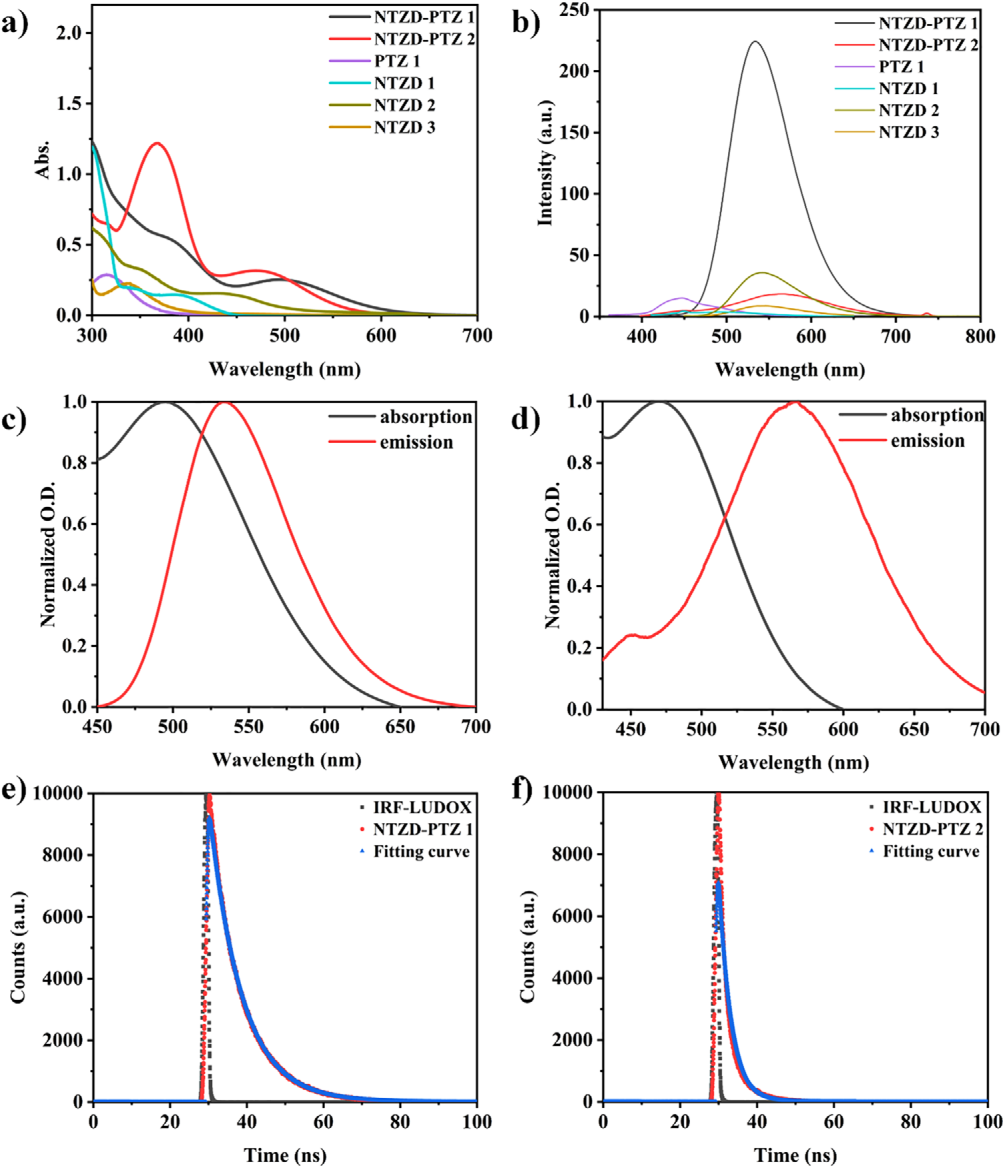
a) UV–vis absorption spectra; b) fluorescence spectra; c) singlet state energy determination of NTZD‐PTZ 1 and d) NTZD‐PTZ 2; and fluorescence lifetime of e) NTZD‐PTZ 1 and f) NTZD‐PTZ 2 in DCM. The concentration of all dyes was set to 2.5 × 10^−5^ M and dissolved in DCM.

The UV‐vis‐light absorption spectra, fluorescence spectra, fluorescence lifetimes, and *E*_S1_ of six dyes and additives are shown in Figure 2, and Figures S1, S2, and S3. These dyes possess a rigid aromatic structure inherently, which makes them exhibit significant absorption in the near‐UV and visible light range (300–500 nm, See Figure 2a),^[^ ^34^ ^]^ with their molar extinction coefficients (*ε*_max_, in visible range) of 10 000 M^−1^cm^−1^(NTZD‐PTZ 1) and 12 800 M^−1^cm^−1^ (NTZD‐PTZ 2) at their maximum absorption wavelength (*λ*_max_) in visible range of 494 and 472 nm respectively. In addition, the UV–vis absorption spectra of EDB and Iod have been measured. As shown in Figure S1, the absorption peaks of EDB and Iod were around 300 and 250 nm, while the absorption peaks of our dyes were basically above 350 nm, so the absorption peaks will not interfere with each other. In addition, the fluorescence spectra of dyes are shown in Figure 2b. Specifically, the fluorescence quantum yield of the dye was measured,^[^ ^25^ ^]^ and the results are given in Table S1. From the Table S1, we can see that NTZD‐PTZ 1 shows the maximum fluorescence quantum yield of 0.85 (anthracene is the reference fluorescence standard). It has the longest fluorescence lifetime among these dyes, measuring 8.51 ns (See Figure 2e, Table 2). The fluorescence lifetimes of other dyes are shown in Figure S2. In particular, the *E*_S1_ can be calculated from the intersection of UV–vis‐light absorption spectra and fluorescence spectra (See Figures 2c,d, and S3) normalized spectra.

**Table 2:** Light absorption and emission characteristics.

PIs	*λ*_max_ in visible range (nm)	*ε*_max_ in visible range (M^−1^cm^−1^)	*ε*_405_ (M^−1^cm^−1^)	*λ*_em_ (nm)	Fluorescence lifetime (ns)
NTZD‐PTZ 1	494	10 000	16 000	533	8.51
NTZD‐PTZ 2	472	12 800	20 400	567	3.20
PTZ 1	320	11 200	280	450	2.89
NTZD 1	400	5600	4800	500	3.75
NTZD 2	440	6000	6000	542	3.75
NTZD 3	335	8800	1000	542	3.72

### Photochemical Properties of Dyes (Steady State Photolysis and ESR Experiments)

**Figure 3:**
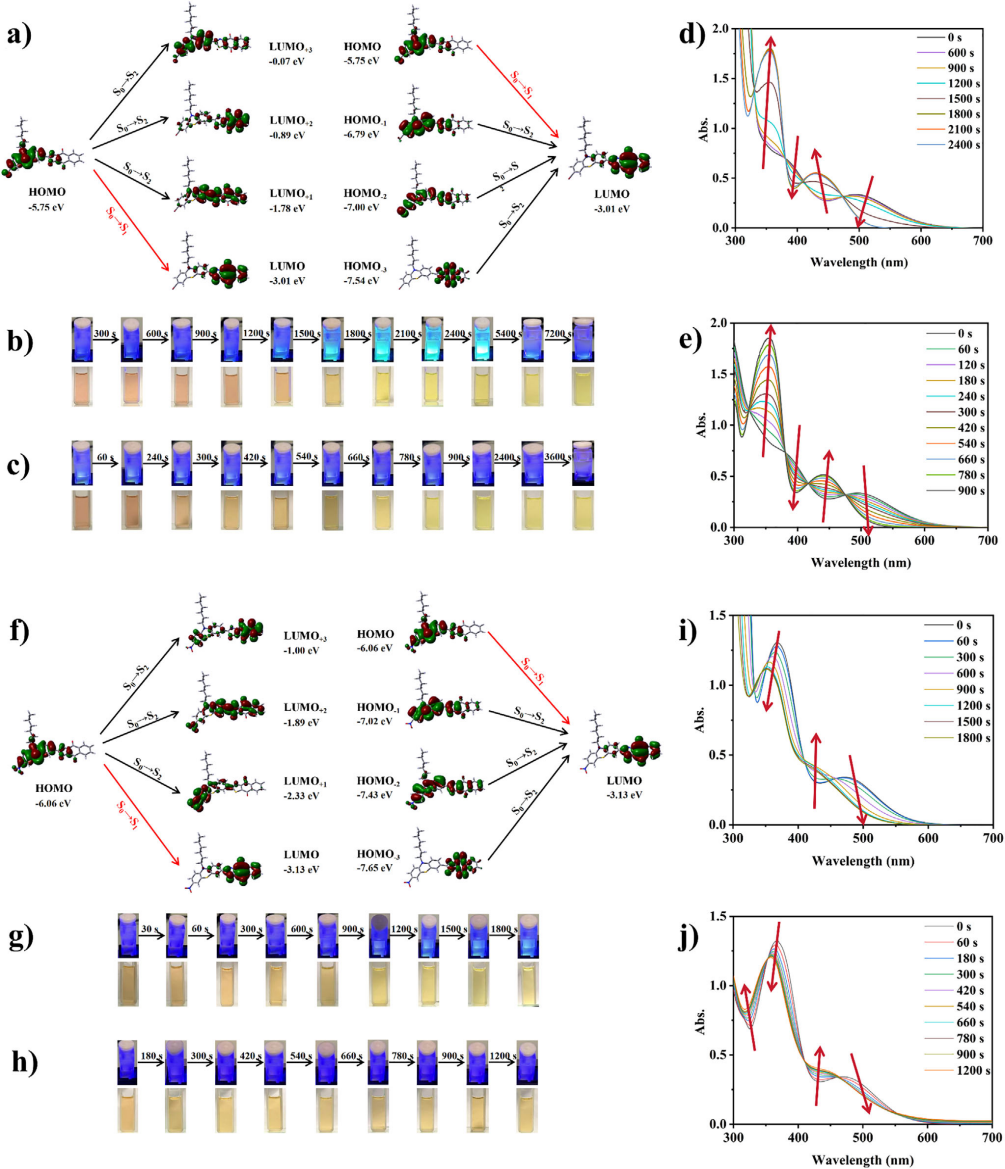
Frontier molecular orbital diagrams of a) NTZD‐PTZ 1. Fluorescence changes of b) NTZD‐PTZ 1/EDB photolysis and c) NTZD‐PTZ 1/Iod photolysis. Steady state photolysis profile of d) NTZD‐PTZ 1/EDB and e) NTZD‐PTZ 1/Iod. Frontier molecular orbital diagrams of f) NTZD‐PTZ 2. Fluorescence changes of g) NTZD‐PTZ 2/EDB photolysis and h) NTZD‐PTZ 2/Iod photolysis. Steady state photolysis profile of i) NTZD‐PTZ 2/EDB and j) NTZD‐PTZ 2/Iod. The light source is LED@405 nm.

The photochemical properties of six dyes were investigated by steady‐state photolysis and ESR experiments. First, the following systems with the highest *ε*_405_ at 405 nm were selected for analysis: NTZD‐PTZ 1/EDB, NTZD‐PTZ 1/Iod, NTZD‐PTZ 2/EDB, and NTZD‐PTZ 2/Iod. Figure 3 shows the electron cloud distribution of all HOMO and LUMO orbitals of NTZD‐PTZ 1 and NTZD‐PTZ 2, photolysis curve and fluorescence changes during photolysis. In the photolysis curve, it is evident that the four systems not only exhibited a degradation performance but also generated new substances when exposed to LED@405 nm light. The photolysis of other dyes is shown in Figures S4, S5, and S6. Compared with the photolysis of dye‐alone system (See Figure S4), the addition of EDB (dye/EDB system) significantly accelerated the photolysis of dyes (See Figure S5). The photolysis of dye/Iod system was slower than that of dye/EDB system (See Figure S6). This also exhibited the occurrence of an electron transfer process between dyes and additives. According to the results of molecular simulation calculation (See Figure 3a,f), NTZD‐PTZ 1 and NTZD‐PTZ 2 had similar molecular structures and similar HOMO and LUMO orbital electron cloud distributions. HOMO values are −5.75 and −6.06 eV respectively; the LUMO values are −3.01 and −3.13 eV respectively. In fact, HOMO is located on the electron‐donating phenothiazine moieties, corresponding, LUMO is located on the electron‐accepting naphtho[2,3‐*d*]thiazole‐4,9‐dione moieties. This is the complete isolation of electron‐donating and electron‐accepting moieties, resulting from the highly twisted large dihedral angle structure. As shown in Figures 3a,f, it is obvious that the clear delocalization of electrons of photoexcited HOMO to LUMO transition corresponded to the charge transfer reaction.^[^ ^33^ ^]^ At the same time, the changes of fluorescence in the photolysis process at different times were recorded (See Figures 3b,c,g,h), especially in the photolysis process of NTZD‐PTZ 1/EDB system (See Figure 3b), the obvious fluorescence enhancement could be observed after 1200 s irradiation at 405 nm. By analyzing the photolysis spectrum of NTZD‐PTZ 1/EDB system (See Figure 3d), an absorption peak at 350 nm was observed, with its intensity suddenly increasing after 1200 s of irradiation at LED@405 nm. We think that this may be because NTZD‐PTZ 1 and EDB produced new fluorescent substances to enhance the fluorescence. These findings highlighted the great consistency between our experimental results and phenomena.

**Figure 4:**
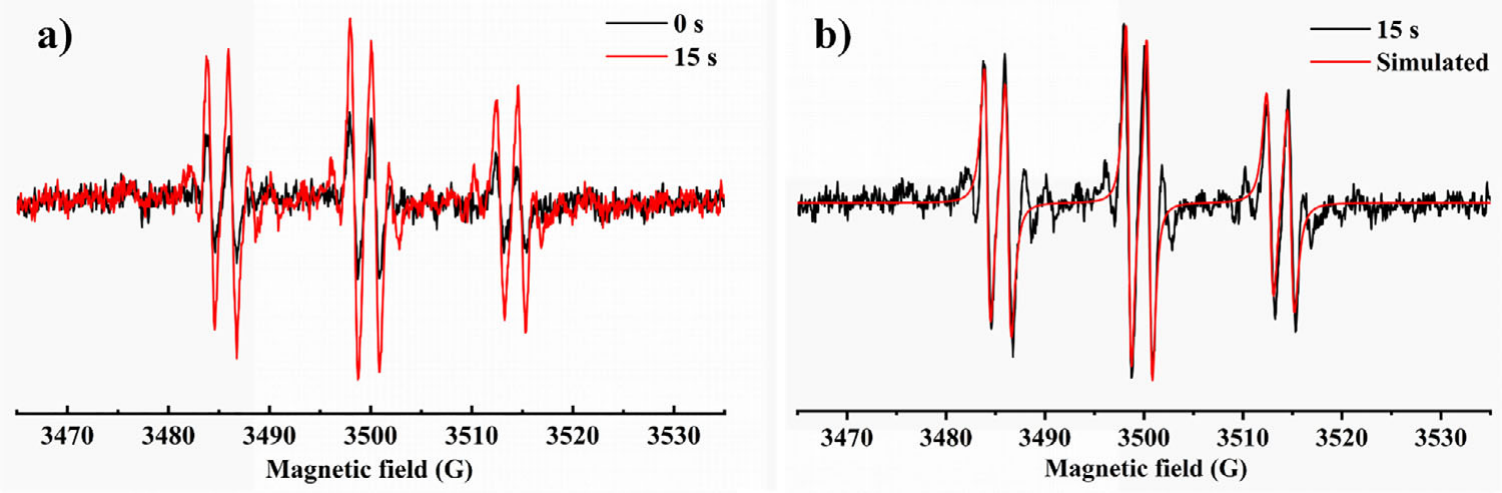
ESR‐ST spectra of the PBN radical adducts (in *tert*‐butylbenzene under nitrogen atmosphere). a) and b) NTZD‐PTZ 1/EDB/Iod under LED@405 nm irradiation.

Then, the ESR spectra of dye/EDB/Iod system irradiated by LED@405 nm were depicted in Figure 4. The NTZD‐PTZ 1/EDB/Iod system were displayed in Figure 4a,b, with hyperfine coupling constants of αN = 14.3 G and αH = 2.1 G (accuracy ± 0.1 G). The calculated hyperfine coupling constants obtained from the system referred to phenyl radicals.^[^ ^34^, ^35^ ^]^ This indicated that this dye possessed remarkable sensitizing capabilities for EDB and Iod.

### Fluorescence Quenching Experiments and Chemical Mechanism in Electron Transfer Reaction for Dye

**Figure 5:**
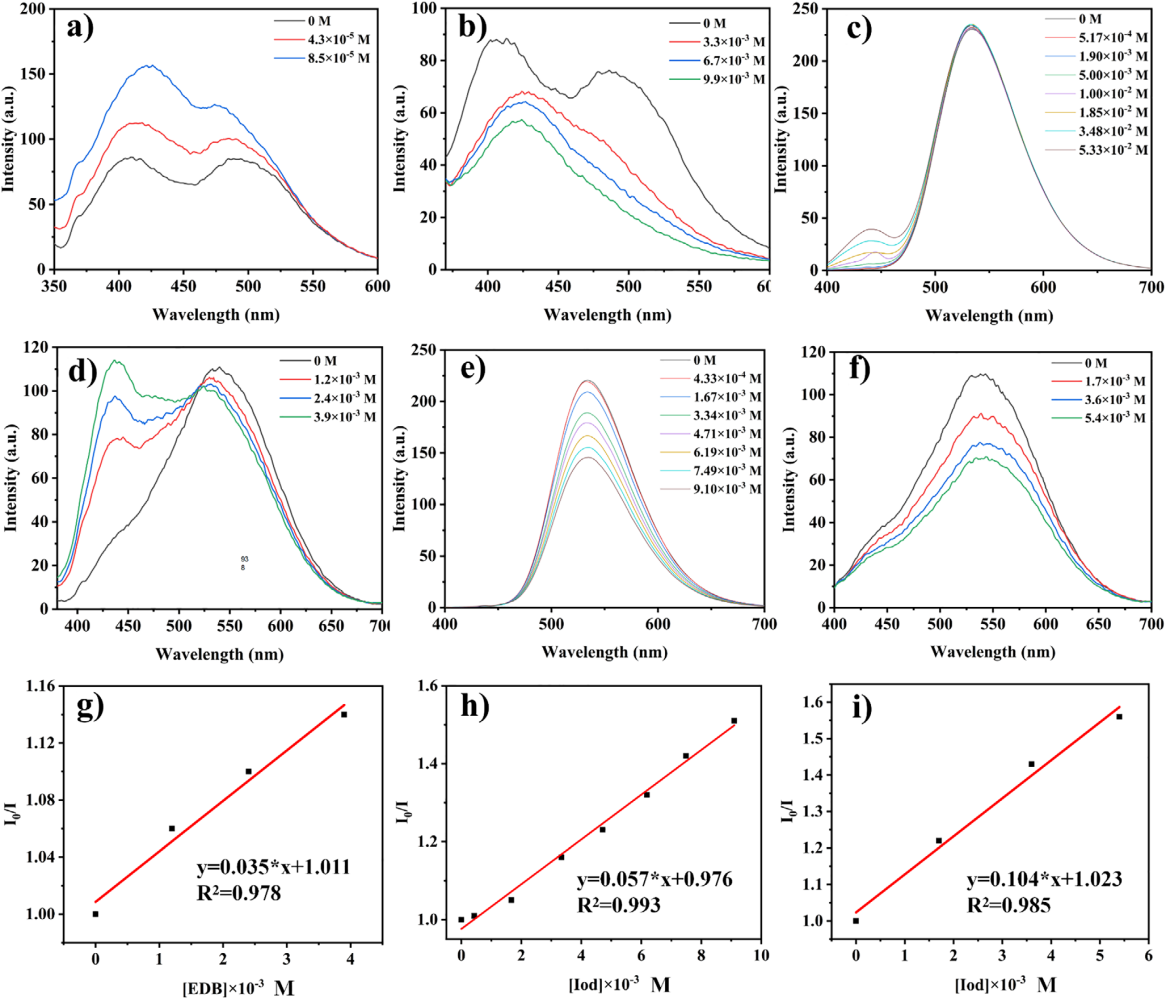
Fluorescence quenching of a) NTZD 1/EDB, b) NTZD 1/Iod, c) NTZD‐PTZ 1/EDB, d) NTZD‐PTZ 2/EDB, e) NTZD‐PTZ 1/Iod, and f) NTZD‐PTZ 2/Iod. Stern−Volmer treatment for fluorescence quenching of g) NTZD‐PTZ 2/EDB, h) NTZD‐PTZ 1/Iod, and i) NTZD‐PTZ 2/Iod, respectively.

Another important method to study electron transfer between dyes and additive is fluorescence quenching experiment. As shown in Figure 5, the fluorescence quenching experiments of dye/EDB and dye/Iod system were carried out in DCM. It can be seen from Figures 5 and S7 that all dyes had different degrees of interaction with EDB and Iod, resulting in changes in fluorescence intensity. The *K*_sv_ between dye and additives were determined by the slope of fluorescence quenching Stern–Volmer treatment (See Figures 5g–i and S7d,e), and the electron transfer quantum yields were calculated by *K*_sv_ (See Table 3). Interestingly, Figures 5a,b show the fluorescence interaction patterns of NTZD 1 with EDB and Iod, respectively. The result indicates that the reaction between NTZD 1 with EDB did not decrease the fluorescence intensity, instead, the fluorescence intensity increased with higher EDB dosage. This may be caused by the production of new fluorescent substances, which was in line with the phenomenon of enhanced fluorescence observed in the process of photolysis. In addition, the obvious oxidation and reduction potentials of dyes were also detected by cyclic voltammetry (See Figure S8). The Δ*G* ^S1^ of electron transfer between dyes and additives (EDB and Iod) were calculated according to the Rehm–Weller equation,^[^ ^29^ ^]^ and the values are listed in Table 3. It can be seen from the Table that Δ*G* ^S1^ of all dyes were negative, this demonstrated that the electron transfer reaction between dyes and additives was feasible.

**Table 3:** Parameters of the chemical mechanisms associated with dyes in DCM.

	NTZD‐PTZ 1	NTZD‐PTZ 2	PTZ 1	NTZD 1	NTZD 2	NTZD 3
*E*_S1_ (eV)	2.36	2.43	3.29	2.81	2.51	2.62
Δ*G* ^S1^_EDB_ (eV)	−0.27	−0.54	–	−0.54	−0.04	−0.01
Δ*G* ^S1^_Iod_ (eV)	−0.34	−0.71	–	−1.57	–	–
E_T1_ (eV)	1.79	1.85	2.61	1.97	1.78	2.28
ΔG^T1^_EDB_ (eV)	0.28	0.67	–	0.30	0.68	0.34
ΔG^T1^_Iod_ (eV)	0.22	−0.04	–	−0.71	–	–
K_sv_ (EDB) s^−1^	–	35	–	–	66	–
K_sv_ (Iod) s^−1^	57	104	–	188	30	–
ϕ_et_ (EDB)^a)^	–	64	–	–	77	–
ϕ_et_ (Iod)^a)^	53	68	–	79	38	–

^a)^ the electron transfer quantum yield is calculated from: ɸ_et _= K_sv_ [additive]/(1 + K_sv_ [additive]); with [additive] the EDB (0.05 M) and Iod (0.02 M) concentration.

**Scheme 3:**
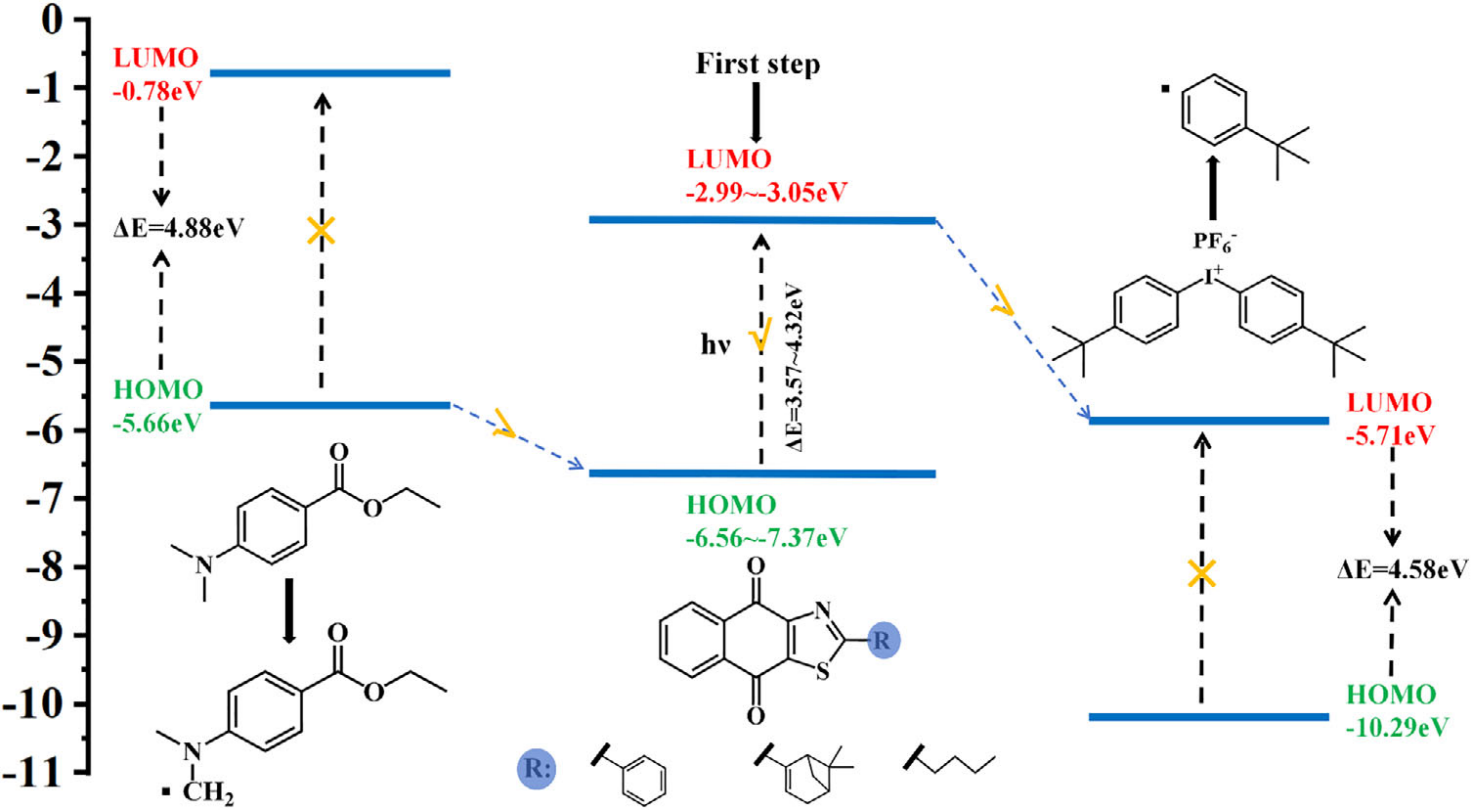
Chemical mechanism of three‐component electron transfer reaction.

Based on the above experimental results and molecular simulations, we proposed a reaction mechanism for the three‐component systems via electron transfer, as shown in Scheme 3. When the dye was excited by light, its HOMO electrons of the ground state transitioned to the excited LUMO orbitals. Then, the electrons in the EDB HOMO level can be easily transferred to the HOMO level of the dye. Similarly, the LOMO level electrons of dyes can be transferred to the LUMO level of Iod. This process demonstrates that the three‐component system can undergo electron transfer reaction with each other to produce free radicals upon light excitation. The feasibility of these electron transfer reactions is theoretically supported and further validated by subsequent experiments.

### Photopolymerization Properties Under LED@405 nm, 450 nm, and Sunlight

**Figure 6:**
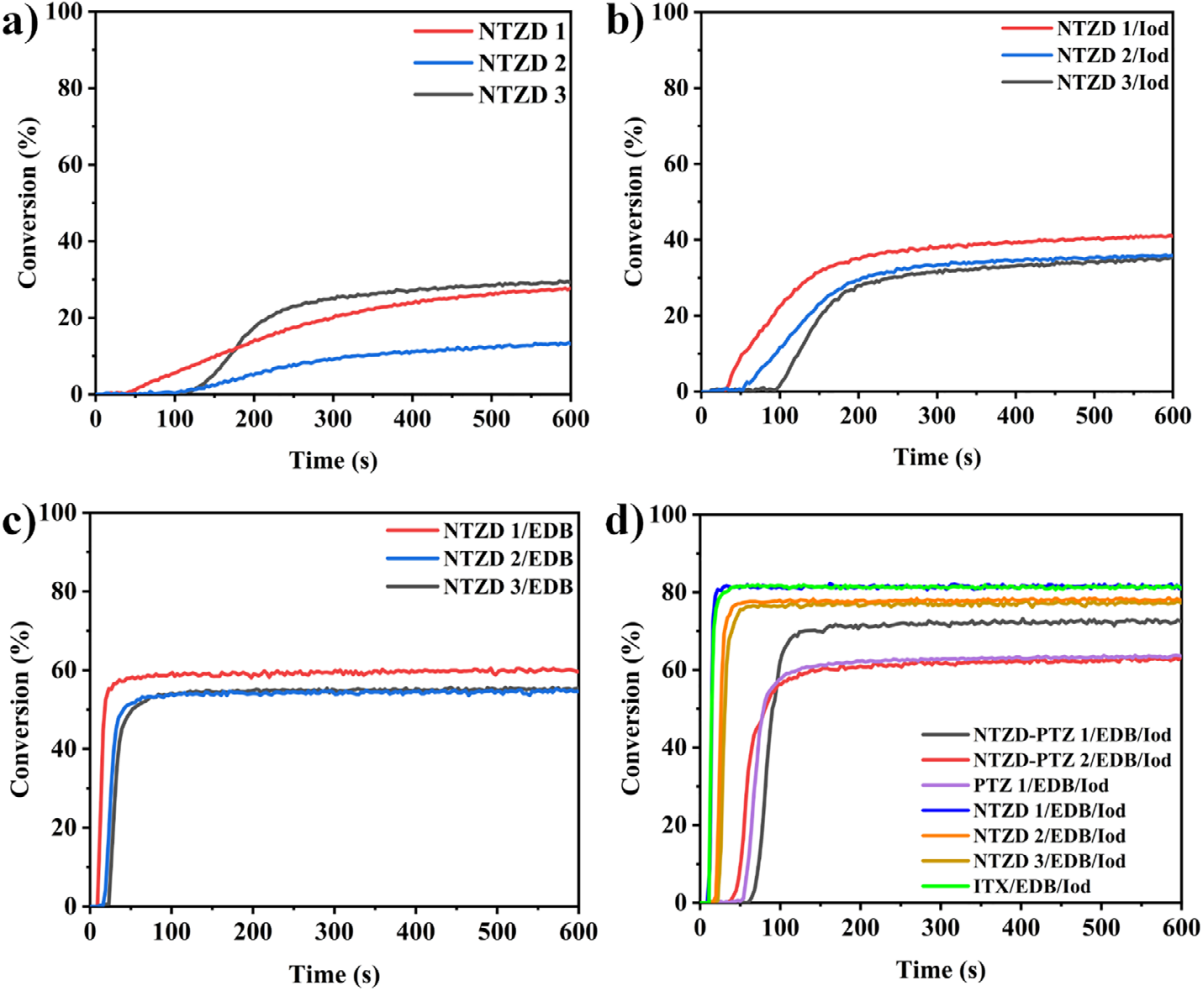
Photopolymerization profiles of TMPTA for thick sample (2 mm) upon exposure to LED@405 nm in the presence of a) dye‐alone 0.01wt%; b) dye/Iod 0.01wt%/1wt%; c) dye/EDB 0.01wt%/1wt%; and d) dye/EDB/Iod 0.01wt%/1wt%/1wt%. The irradiation starts at *t* = 10 s.

**Figure 7:**
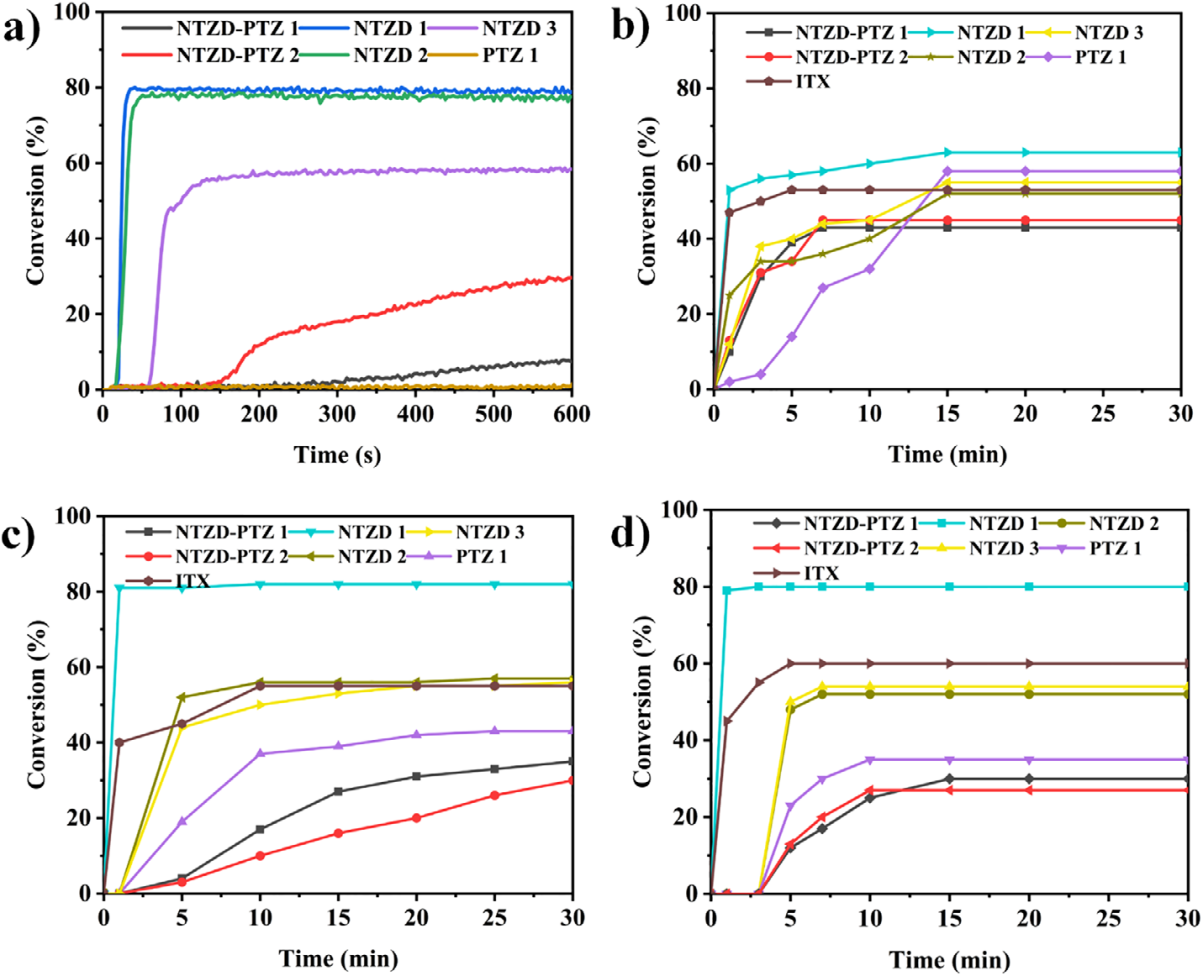
Photopolymerization profiles of TMPTA for thick sample (2 mm) upon exposure to LED@450 nm in the presence of a) dye/EDB/Iod 0.01% wt/1%wt/1%wt. The irradiation starts at *t* = 10 s. Photopolymerization profiles of TMPTA under sunlight irradiation. b) Thick sample (A cylinder with a diameter of 8 mm and a thickness of 2 mm) dye/EDB/Iod 0.01wt%/1wt%/1wt%. c) Thick sample (A cylinder with a diameter of 14 mm and a thickness of 4 mm) dye/EDB/Iod 0.01wt%/1wt%/1wt% and d) thick sample (A cylinder with a diameter of 14 mm and a thickness of 2 mm) dye/EDB/Iod 0.01% wt/1%wt/1%wt.

Photopolymerization capacity of dye‐initiated TMPTA was evaluated using RT‐FTIR, with the resulting polymerization profiles shown in Figure 6. For comparison, the photopolymerization efficiency of one‐component systems, two‐component systems, and three‐component systems were tested for thick samples irradiated by LED@405 nm. The concentrations of the additive EDB/Iod were fixed at 1 wt%. In the one‐component system (See Figure 6a), we can see that NTZD 1, 2, and 3 can initiate the polymerization, which was consistent with the steady‐state photolysis experiment of dye‐alone (See Figure S4). However, the single dye did not show good initiation performance, and the FCs of acrylate were lower than 30%. In the two‐component system (See Figure 6b,c), the FCs of both dye/Iod and dye/EDB systems were improved, and the maximum FCs was close to 60% (See Figure 6c NTZD 1). In the three‐component system (See Figure 6d), all dyes can initiate the deep polymerization of monomers, and the FCs of TMPTA initiated by NTZD 1/EDB/Iod system reached 82%, which showed similar photoinitiation capacity to commercial initiator ITX. It was worth noting that in the three‐component system, the FCs of TMPTA initiated by NTZD‐PTZ 1 and NTZD‐PTZ 2, which contained phenothiazine and naphtho[2,3‐*d*]thiazole‐4,9‐dione, were only 70% and 60% respectively, which may be because the internal filtering effect caused by their high molar extinction coefficient hindered the penetration of light in depth. The lower concentration of the initiator can also have an influence. The photoinitiation efficiency of three‐component system was better than that of two‐component system and one‐component system, highlighting the crucial role of dyes in PISs. Dyes can facilitate electron transfer and improve photoinitiation efficiency. The results show that the dyes investigated in this study exhibit exceptional photoinitiation capabilities even at ultralow concentration (0.01 wt%). In addition, the photoinitiation efficiency of these dyes under 450 nm blue light was also tested. The results are shown in Figure 7a. We can see that the conversion of NTZD 1 with the best performance can still reach 80% under low power blue light. Notably, the commercial initiator ITX does not effectively initiate polymerization under this blue light.

Unlike artificial light sources, sunlight offers numerous advantages and is the ideal light source for photopolymerization. Although sunlight has a broader emission spectrum, its intensity is relatively low (2 mW.cm^−2^).^[^ ^36^ ^]^ According to the analysis of ASTM G173‐03 reference spectrum, the sunlight radiation in the range of 280–400 nm is in the range of 0.5–1.4 mW.cm^−2^, and the photopolymerization time could be elongated from several hours to several days^[^ ^31^, ^37^ ^]^

Given the excellent light absorption properties of the dyes in this study, samples of varying thicknesses were polymerized under sunlight, as depicted in Figure 7b,c,d. These figures illustrate the polymerization profiles of cylindrical samples: one with a thickness of 2 mm and a diameter of 8 mm, another with a thickness of 4 mm and a diameter of 14 mm, and a third with a thickness of 2 mm and a diameter of 14 mm. Remarkably, high FCs were achieved within 10–15 min across all samples with NTZD 1 as the PI, outperforming the commercial initiator ITX in each case. Interestingly, the FCs varied with sample dimensions. The 14 mm diameter samples, regardless of thickness (2 or 4 mm), achieved approximately 81% conversion (Figure 7c,d), whereas the 8 mm diameter sample (2 mm thick) reached only 55% (Figure 7b). This suggests that samples with larger surface areas can receive greater sunlight exposure, enhancing the activation of the PISs and thereby improving the efficiency of monomer polymerization. The performance of NTZD 1 in sunlight surpassed that of many previously reported phenothiazine and naphthoquinone derivative dyes used under LED@405 nm irradiation.^[^ ^32^ ^]^ The exceptional photoinitiation capacity of NTZD 1 underscores its significant potential in sunlight‐induced polymerization. The great geometrical arrangement, recognizable by the representation of the HOMO orbital (Figure 1) could be a decisive factor that overcompensates for the lower absorption.

### Storage Stability of the Formulations

**Figure 8:**
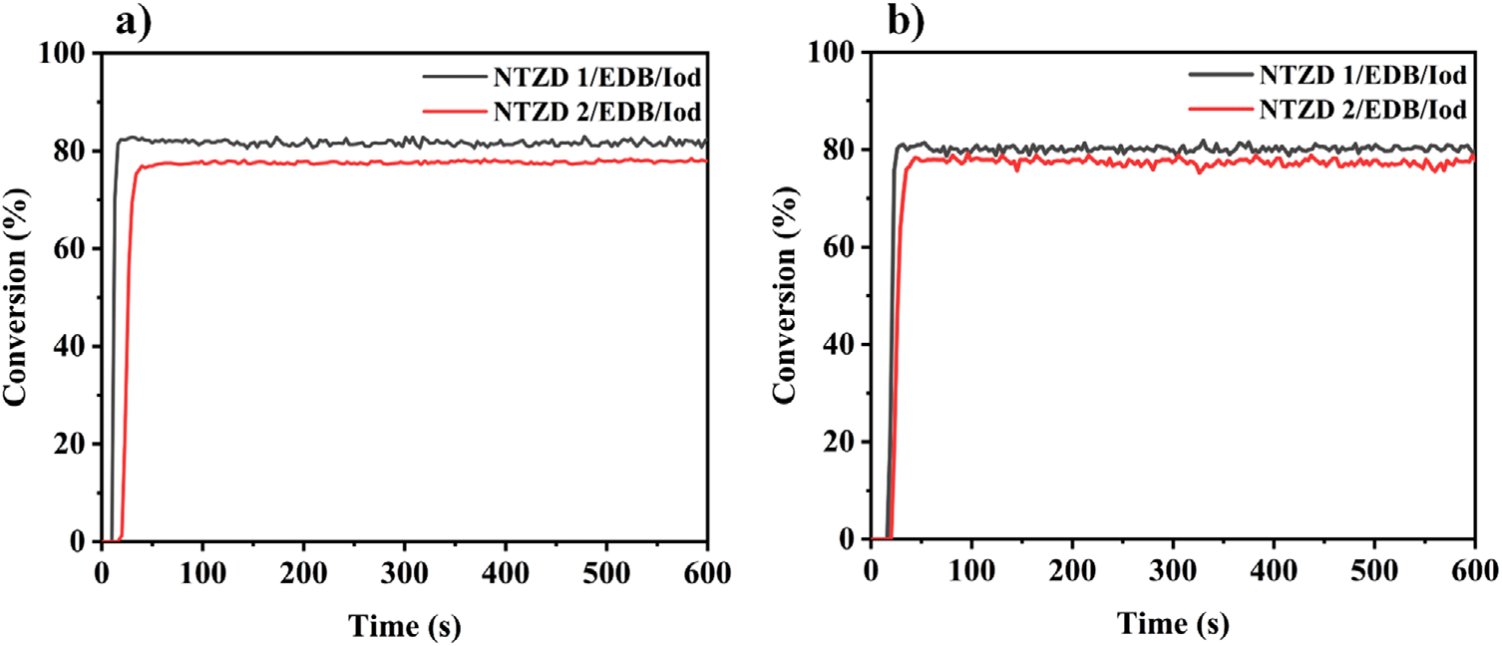
Photopolymerization kinetics for thick sample (about 2 mm) of TMPTA after two weeks. a) under LED@405 nm irradiation; b) under LED@450 nm irradiation. Initiated by dye/EDB/Iod 0.01% wt/1%wt/1%wt. The irradiation starts at *t* = 10 s.

The storage stability of the formulations containing the dyes was investigated. The photopolymerization kinetics of two most active three‐component formulations (NTZD 1/EDB/Iod and NTZD 2/EDB/Iod) were evaluated after being stored away from light for two weeks (Figure 8). When compared to the polymerization profiles from two weeks prior (Figures 6d and 7a), the FCs of the two formulations under 405 and 450 nm irradiation showed almost no change. These results suggest that the dyes maintained stability in the formulations when protected from light, which is advantageous for transportation and practical application.

### Comparisons with PIs from Previous Studies

A detailed comparison with dye‐based PISs previously reported in the literature highlighted the exceptional performance of the dyes investigated in this study. Table 4 summarizes the properties of several dyes, including the top candidate for TMPTA free radical photopolymerization identified in previous studies, where polymerization experiments consistently employed a dye/EDB/Iod concentration of 0.1wt%/2wt%/2wt%. In contrast, this study utilized a reduced concentration of dye/EDB/Iod at 0.01 wt%/1 wt%/1 wt%. Despite this substantial 10‐fold decrease in dye concentration, the formulations in this study achieved high monomer conversions (82%). Additionally, the exposure time to sunlight was significantly reduced, with NTZD 1 demonstrating notable monomer conversion (81%) after just 30 s, compared to 30 min in earlier studies. Moreover, under blue light polymerization at LED@450 nm, NTZD 1 maintained high monomer conversion (80%). Based on these results and to the best of our knowledge, NTZD 1 stood out as one of the first sunlight PIs capable of achieving high monomer conversion with such low concentrations in approximately 1 min of sunlight exposure. This positioned NTZD 1 as a leader among solar photoinitiators for efficient and rapid polymerization under sunlight. In the literature, a three‐component PIS (dye/EDB/Iod 0.1wt%/2wt%/2wt%) similar to that in this study has been successfully applied only to the DLW 3D printing experiment. By contrast, the three‐component system based on NTZD 1 in this study excelled across three 3D printing technologies (i.e., DLW, LCD, and DLP) as demonstrated below. The ability of this system to perform effectively in three distinct 3D printing technologies demonstrated the versatility of the dyes developed here, enabling their application across a broad range of 3D printing technologies and highlighting their superior performance.

**Table 4:** Acrylate function conversions obtained during free radical polymerization of TMPTA at 405 nm and sunlight using three‐component PISs dye/Iod/EDB (0.1wt%/2wt%/2wt%).

	Dyes	Absorption properties	Final acrylate function conversions	Print type
**Dye1** ^[^ ^38^ ^]^	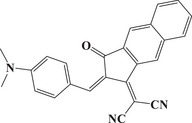	*λ*_max_ = 582 nm *ε*_max_ = 24 872 M^−1^.cm^−1^ *ε*_405nm _= 6870 M^−1^.cm^−1^	∼80% (LED@405 nm, 400 s)	Only DLW
**Dye2** ^[^ ^38^ ^]^	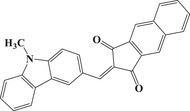	*λ*_max_ = 447 nm *ε*_max_ = 49 650 M^−1^.cm^−1^ *ε*_405nm _= 22 910 M^−1^.cm^−1^	∼76% (LED@405 nm, 400 s)	Only DLW
**Dye3** ^[^ ^38^ ^]^	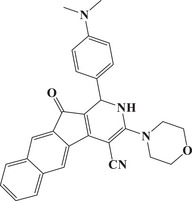	*λ*_max_ = 491 nm *ε*_max_ = 5330 M^−1^.cm^−1^ *ε*_405nm _= 3340 M^−1^.cm^−^	∼91% (LED@405 nm, 400 s); ∼88% (sunlight, 30 min)	Only DLW
**Dye4** ^[^ ^39^ ^]^	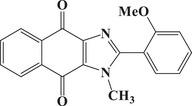	*λ*_max_ = 380 nm *ε*_max_ = 1300 M^−1^.cm^−1^ *ε*_405nm _= 1000 M^−1^.cm^−1^	∼80% (LED@405 nm, 400 s)	Only DLW
**Dye5** ^[^ ^39^ ^]^	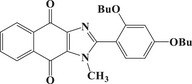	*λ*_max_ = 401 nm *ε*_max_ = 1460 M^−1^.cm^−1^ *ε*_405nm _= 1450 M^−1^.cm^−1^	∼80% (LED@405 nm, 400 s)	Only DLW
**Dye6** ^[^ ^39^ ^]^	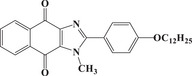	*λ*_max_ = 405 nm *ε*_max_ = 1600 M^−1^.cm^−1^ *ε*_405nm _= 1600 M^−1^.cm^−1^	∼80% (LED@405 nm, 400 s)	Only DLW

### Application of 3D Printing Experiments

**Figure 9:**
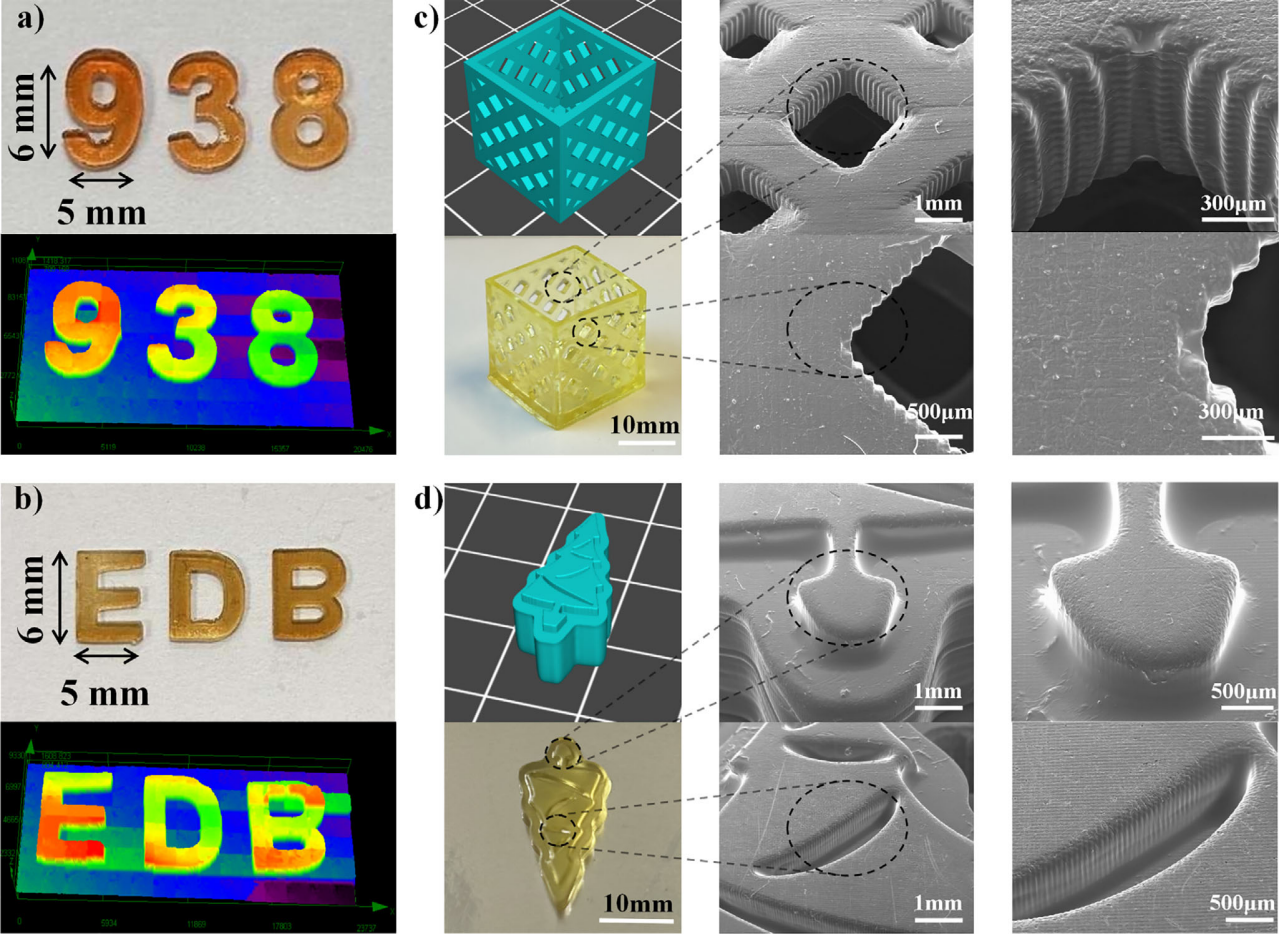
Demonstration of high‐precision structure construction of different 3D printing technologies. a) and b) 3D numbers and letters printed by DLW printing technique and numerical optical microscopy characterization, c) The “hollow cube” printed by LCD printing technique and SEM characterization. d) The “Christmas tree” printed by DLP printing technique and SEM characterization.

In DLW printing, NTZD‐PTZ 1/EDB/Iod (0.05 wt%/1 wt%/1 wt%) and NTZD‐PTZ 2/EDB/Iod (0.05 wt%/1 wt%/1 wt%) produced 3D patterns of the number “938” and the letter “EDB”, respectively. As shown in Figure 9a,b, the outlines of the pattern were clearly defined. In LCD printing, NTZD 1/EDB/Iod (0.05 wt%/1 wt%/1 wt%) was used to make a “hollow cube” object. As shown in Figure 9c, this 3D hollow cube model of complex structure model was printed flawlessly. SEM characterization revealed that the printed cube surface and grid exhibited excellent resolution. In DLP printing, NTZD 1/EDB/Iod (0.05 wt%/1 wt%/1 wt%) was used to print “Christmas tree” object, as shown in Figure 9d, and the surface of the objects also showed excellent resolution. The SEM images further confirmed that smooth surfaces and excellent spatial resolution were achieved in the fabrication of these 3D objects, with all items exhibiting satisfactory accuracy across various sizes. In addition, in DLP printing, we also successfully printed the “hollow pattern box” object with NTZD1/EDB/Iod (0.05 wt%/1 wt%/1 wt%) system (Figure S9). The pattern was exquisite and the outline of the pattern was clearly visible, which further demonstrated the potential of the developed photoinitiator for 3D printing.

## Conclusion

In conclusion, we reported five dyes with D‐π‐A structures that effectively facilitated electron transfer reactions with additives due to their structural specificity, i.e., the significant separation between electron‐withdrawing and electron‐releasing units. We proposed a chemical mechanism for the electron transfer reaction in three‐component system. The phenyl modified NTZD 1 three‐component system with EDB as amine and iodonium salt exhibited the best photopolymerization capacity under LED@405 nm, achieving a final conversion of 82% for acrylate double bonds of TMPTA, and 81% under sunlight within just 30 s, highlighting its excellent photoinitiation capacity. In addition, the optimal 3D printing precursor formulations were suitable for various 3D printing technologies (i.e., DLW, DLP, and LCD). The resulting high‐precision 3D printed objects further validated the excellent photoinitiation capacity of the dyes. Given the successful application of the D‐π‐A dyes designed in this study of photopolymerization in 3D printing, NTZD 1 was expected to serve as an effective photoinitiator. Thus, this work offered a valuable approach to the molecular design of photosensitive formulations with potential applications in 3D printing. The findings from this research could significantly contribute to the advancement of 3D printing applications across diverse fields, including biomedical engineering, materials science, and custom manufacturing.

## Conflict of Interests

The authors declare no conflict of interest.

## Supporting information



Supporting Information

## Data Availability

The data that support the findings of this study are available from the corresponding author upon reasonable request.
