# Supplementary material for: Donor‐π‐Acceptor Photoinitiators for High‐Efficiency Visible LED and Sunlight Polymerization and High‐Precision 3D Printing
Source: Angew Chem Int Ed Engl. 2025 May 2;64(27):e202425198. doi: 10.1002/anie.202425198 (PMC12207365; doi:10.1002/anie.202425198)
Supplement: Supplementary file 1 — Supporting Information [file ANIE-64-e202425198-s001.docx]

**Supporting Information**

**Donor-π-Acceptor Photoinitiators for High-Efficiency Visible LED and Sunlight Polymerization and High-Precision 3D Printing**

**Ji Feng ^a,b^, Tong Gao ^a,b^, Fabrice Morlet-Savary ^a,b^, Michael Schmitt ^a,b^, Celine Dietlin ^a,b^, Jing Zhang ^c^, Xiaotong Peng ^d^, Pu Xiao ^d^*, Frédéric Dumur ^e^*, and Jacques Lalevée ^a,b^***

^a^ Université de Haute-Alsace, CNRS, IS2M UMR7361, F-68100 Mulhouse, France.

^b^ Université de Strasbourg, France.

^c^ Future Industries Institute, University of South Australia, Mawson Lakes, SA 5095, Australia.

^d^ State Key Laboratory of High-Performance Ceramics and Superfine Microstructure, Shanghai Institute of Ceramics, Chinese Academy of Sciences, Shanghai 200050, P. R. China.

^e^ Aix Marseille Univ, CNRS, ICR, UMR 7273, F-13397 Marseille, France.

E-mail address: jacques.lalevee@uha.fr (J. L.); frederic.dumur@univ-amu.fr (F.D.); p.xiao@mail.sic.ac.cn (P.X.)

**Table S1.** Absorbance, fluorescence spectral area and fluorescence quantum yield.

|  | **NTZD-PTZ 1** | **NTZD-PTZ 2** | **NTZD 1** | **NTZD 2** | **NTZD 3** | **PTZ 1** |
| --- | --- | --- | --- | --- | --- | --- |
| **UV-Vis Abs.** | 0.08 | 0.09 | 0.09 | 0.07 | 0.07 | 0.10 |
| **Fluorescent area** | 5393 | 2623 | 2461 | 2544 | 2721 | 1241 |
| **Fluorescence quantum yield** | 0.85 | 0.36 | 0.34 | 0.45 | 0.48 | 0.22 |

**
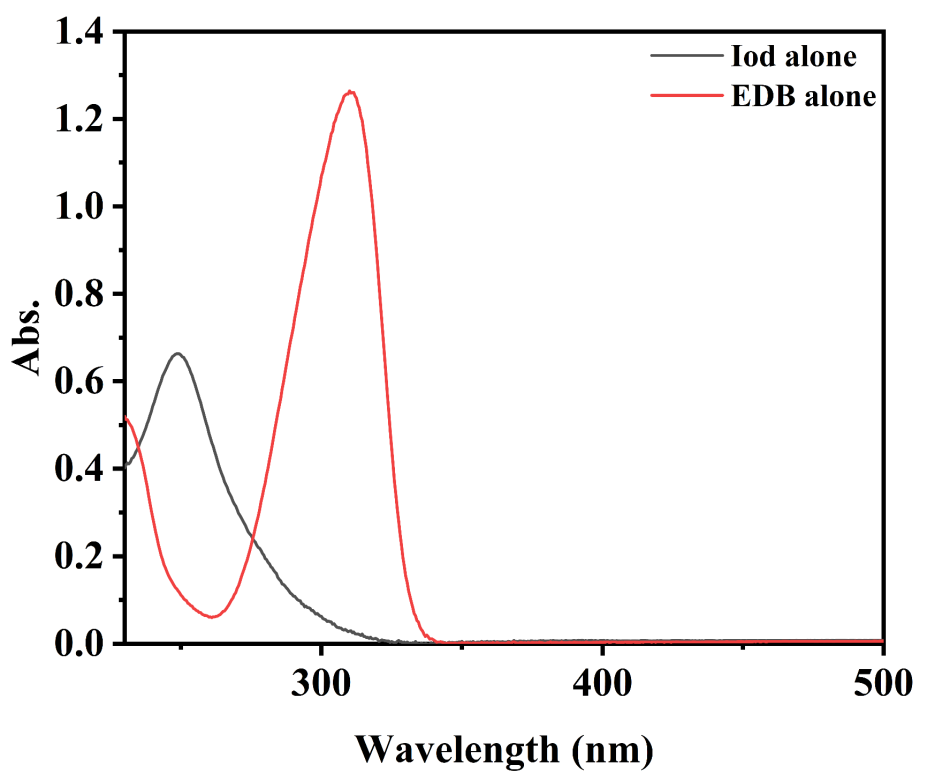
**

**Figure S1.** UV-vis spectra of EDB alone and Iod alone in DCM. The concentrations of EDB and Iod were set to 5×10^-5^ M.

**
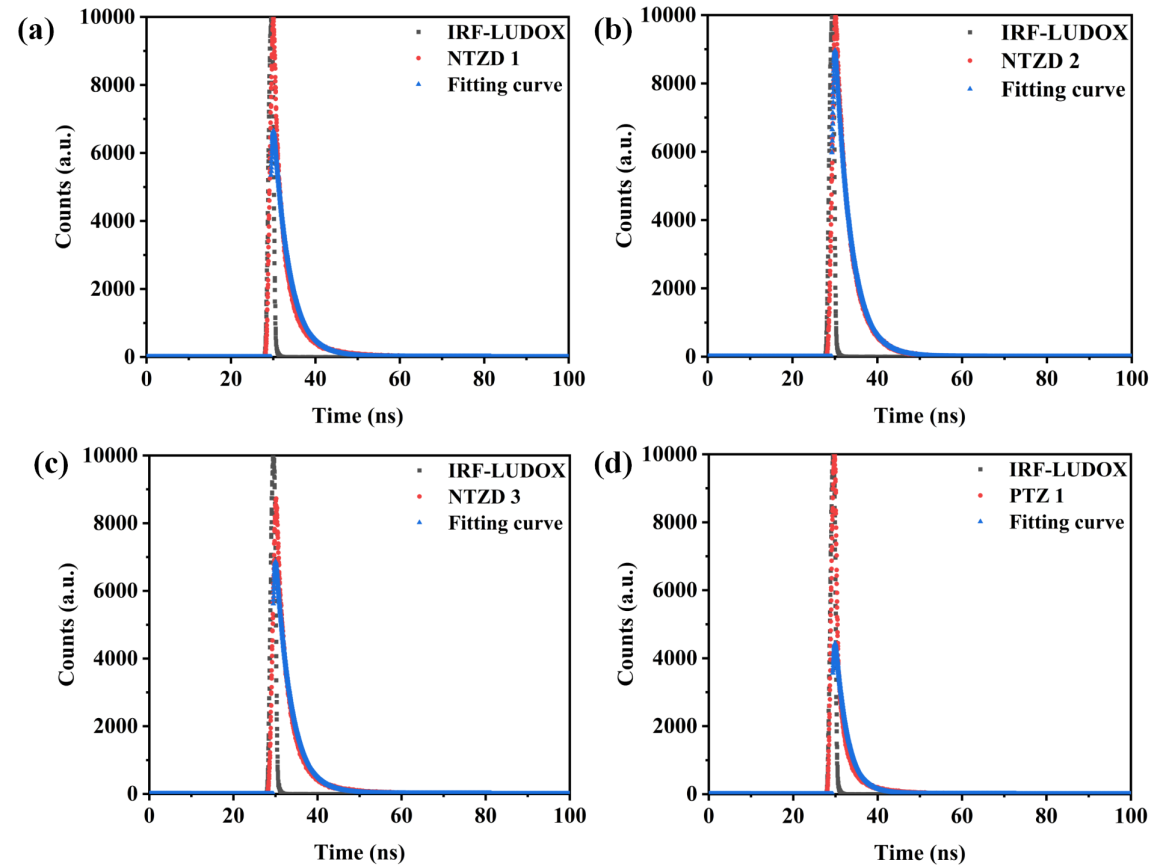
**

**Figure S2.** Fluorescence lifetimes of (a) NTZD 1, (b) NTZD 2 (c) NTZD 3, (d) PTZ 1.

**
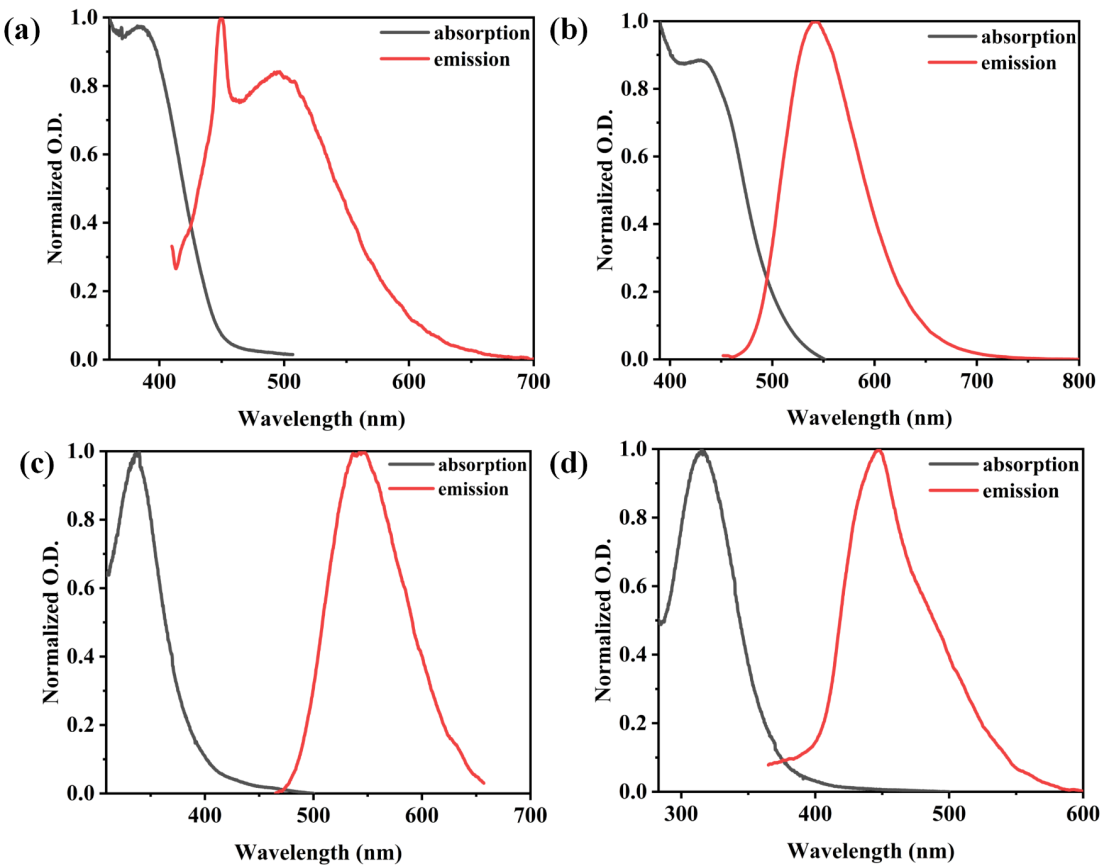
**

**Figure S3.** Singlet energy (a) NTZD 1, (b) NTZD 2 (c) NTZD 3, (d) PTZ 1.

**
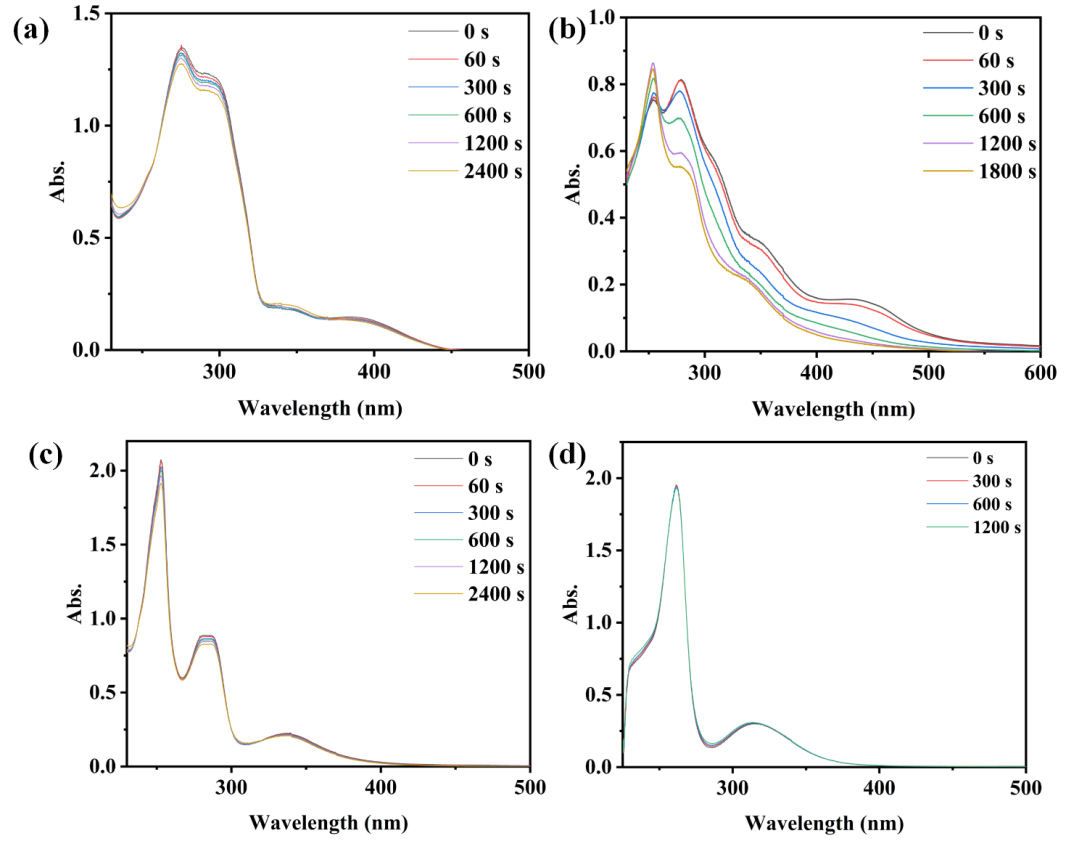
**

**Figure S4.** Steady state photolysis of dye-alone (2.5×10^-5^ M) in DCM under LED@405 nm. (a) NTZD 1, (b) NTZD 2, (c) NTZD 3, (d) PTZ 1.

**
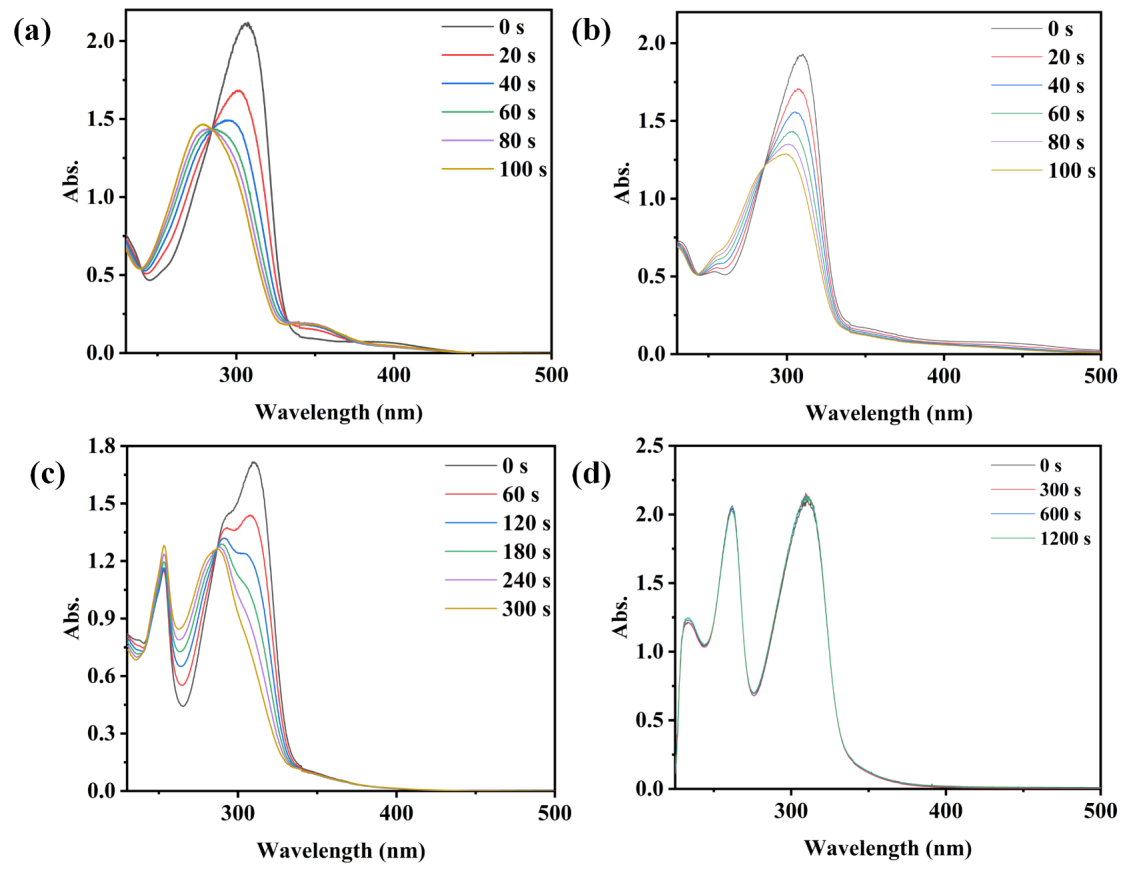
**

**Figure S5.** Steady state photolysis of dye (2.5×10^-5^ M)/EDB (5×10^-5^ M) in DCM under LED@405 nm. (a) NTZD 1, (b) NTZD 2, (c) NTZD 3, (d) PTZ 1.

**
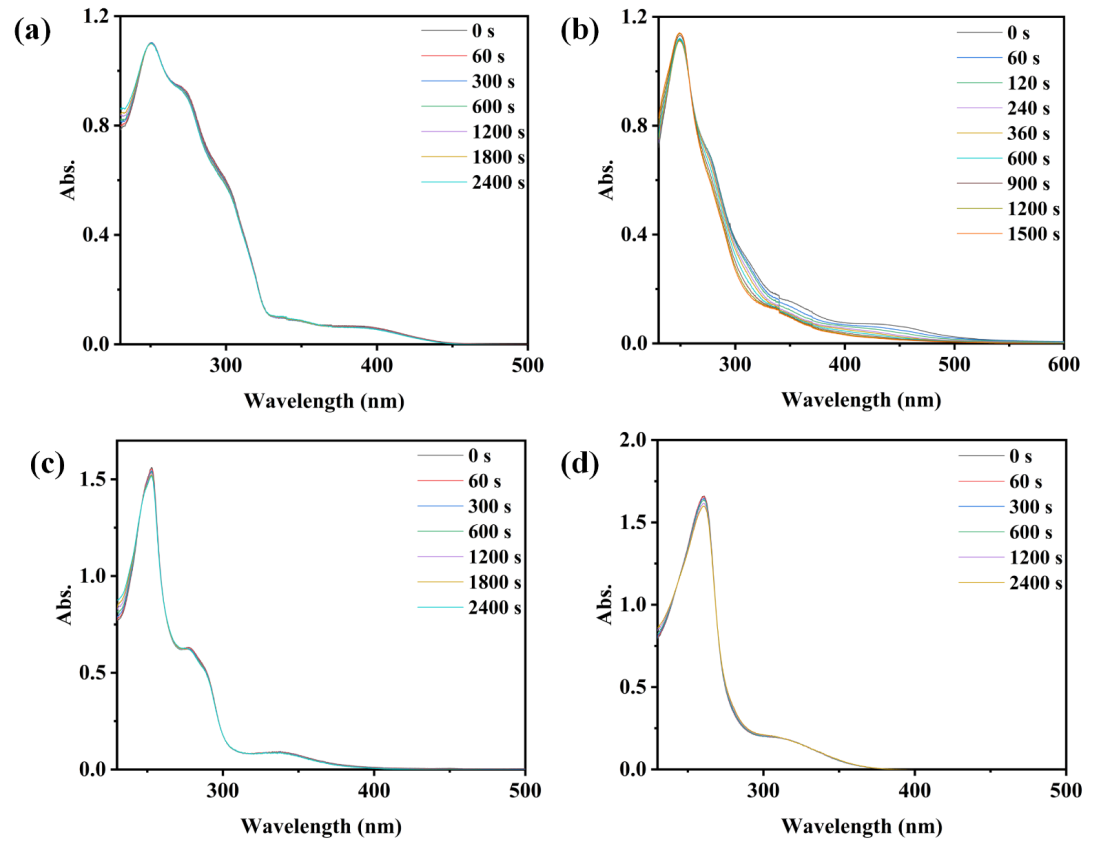
**

**Figure S6.** Steady state photolysis of dye (2.5×10^-5^ M)/Iod (5×10^-5^ M) in DCM under LED@405 nm. (a) NTZD 1, (b) NTZD 2, (c) NTZD 3, (d) PTZ 1.

**
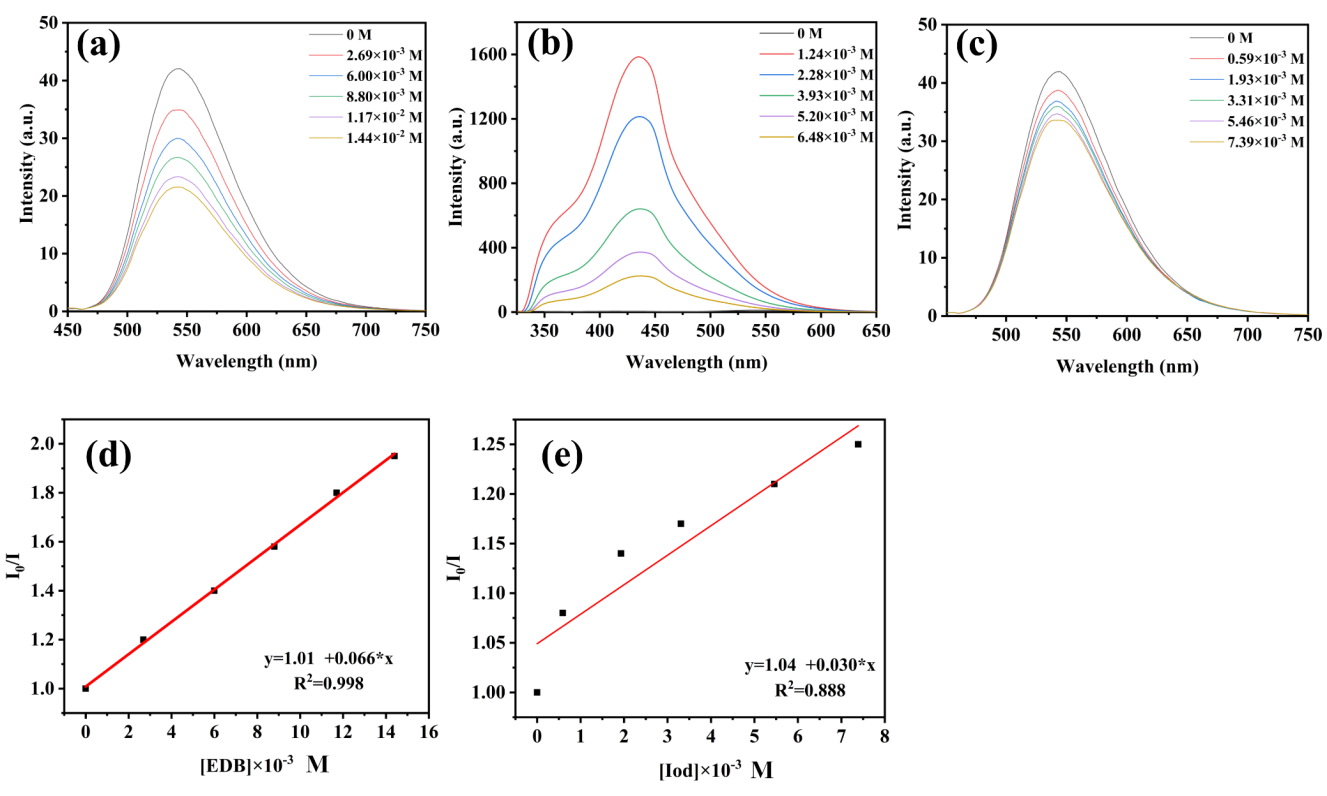
**

**Figure S7.** Fluorescence quenching of (a) NTZD 2/EDB, (b) NTZD 3/EDB (c) NTZD 2/ Iod. Stern − Volmer treatment for fluorescence quenching of (d) NTZD 2/EDB and (e) NTZD 2/Iod, respectively.


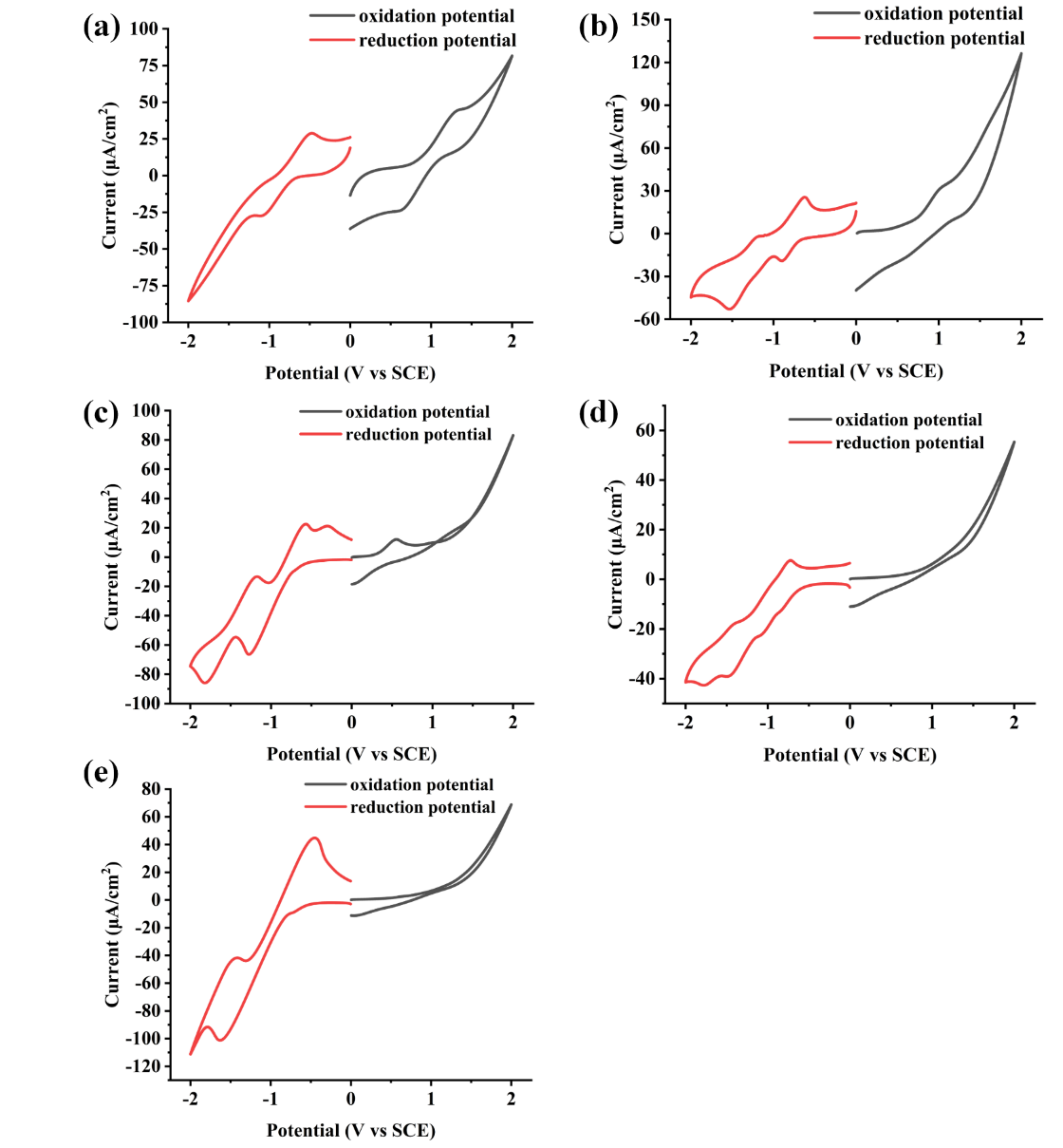


**Figure S8.** Cyclic voltammetry of electrochemical reactions in DCM solvent against saturated calomel electrode (SCE) under N_2_ saturated solution. (a) NTZD-PTZ 1, (b) NTZD-PTZ 2, (c) NTZD 1, (d) NTZD 2, (e) NTZD 3.


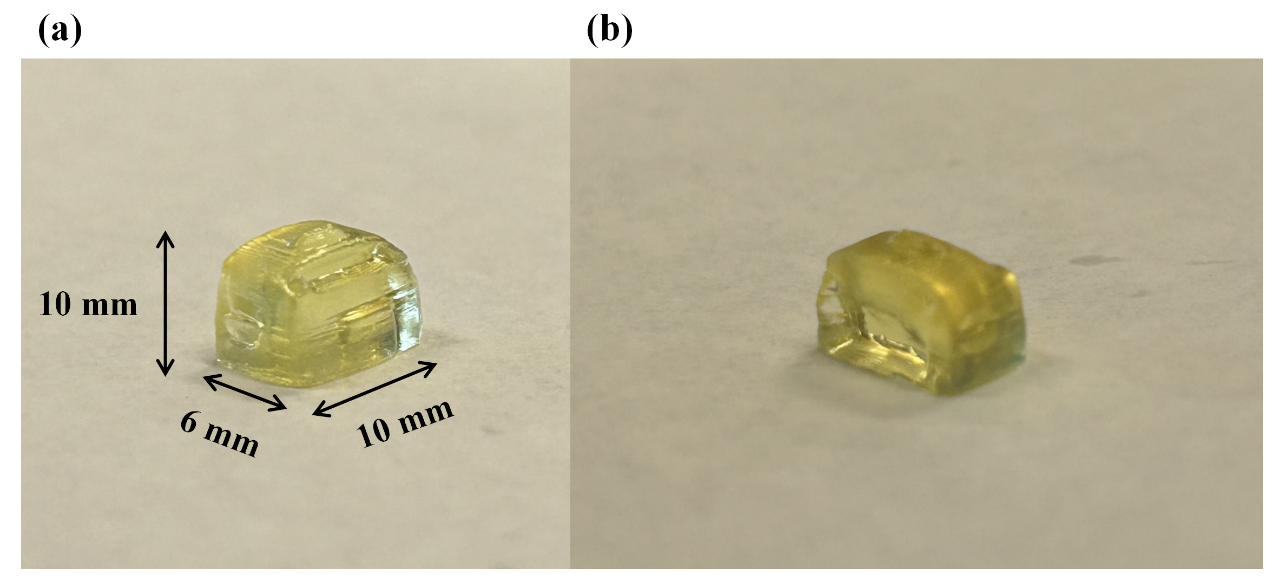


**Figure S9.** Demonstration of high-precision structure of 3D printing technologies. (a) and (b) the " Hollow patterned box" printed by DLP printing technique.

**General information**

All reagents and solvents were purchased from different suppliers (Aldrich, TCI Europe, VWR, Carlo Erba, etc…) and used as received without further purification. ^1^H and ^13^C NMR spectra were determined at room temperature in 5 mm o.d. tubes on a Bruker Avance 400 MHz from BioCis at Cergy Paris University: ^1^H (400 MHz) and ^13^C (100 MHz). All ^1^H chemical shifts were referenced to the solvent peak CDCl_3_ (7.26 ppm), DMSO-d_6_ (2.49 ppm) and the ^13^C chemical shifts were referenced to the solvent peak CDCl_3_ (77.0 ppm).

Synthesis of 2-butylnaphtho[2,3-*d*]thiazole-4,9-dione

A mixture of 2-amino-1,4-naphthoquinone (3.5 g, 20 mmol, M = 173.17 g/mol), valeraldehyde (2.07 g, 2.55 mL, 24 mmol, M = 86.13 g/mol, d = 0.81), S_8_ (1.9 g, 60 mmol, M = 32.07 g/mol) and NaHCO_3_ (1.7 g, 20 mmol, M = 84.01 g/mol) in DMF (50 mL) were stirred at 100°C overnight. The solution was poured into water (50 mL) and extracted with ethyl acetate. The organic phase was washed several times with water. The combined organic layer was dried with anhydrous magnesium sulfate and the solvent was removed with a rotary evaporator. The residue was purified by column chromatography (SiO_2_) using DCM as the eluent. For a higher purity, addition of diethyl ether precipitated a light brown solid that was filtered off, washed several times with ether and dried under vacuum (4.12 g, 76% yield).

^1^H NMR (400 MHz, CDCl_3_) δ 8.41 – 8.26 (m, 1H), 8.26 – 8.15 (m, 1H), 7.79 (t, *J* = 5.3 Hz, 2H), 3.17 (t, *J* = 7.8 Hz, 2H), 1.86 (q, *J* = 7.7 Hz, 2H), 1.47 (q, *J* = 7.5 Hz, 2H), 0.97 (t, *J* = 7.4 Hz, 3H)

^13^C NMR (101 MHz, CDCl_3_) δ 180.19, 178.50, 178.00, 154.43, 141.93, 134.48, 134.08, 133.14, 132.75, 127.89, 127.06, 34.26, 32.10, 22.35, 13.83

^1^H NMR spectrum of 2-butylnaphtho[2,3-*d*]thiazole-4,9-dione

^13^C NMR spectrum of 2-butylnaphtho[2,3-*d*]thiazole-4,9-dione

Synthesis of 2-phenylnaphtho[2,3-d]thiazole-4,9-dione

A mixture of 2-amino-1,4-naphthoquinone (3.5 g, 20 mmol, M = 173.17 g/mol), benzaldehyde (2.55 g, 2.44 mL, 24 mmol, M = 106.12 g/mol, d = 1.044), S_8_ (1.9 g, 60 mmol, M = 32.07 g/mol) and NaHCO_3_ (1.7 g, 20 mmol, M = 84.01 g/mol) in DMF (50 mL) were stirred at 100°C overnight. The solution was poured into water (50 mL) and extracted with ethyl acetate. The organic phase was washed several times with water. The combined organic layer was dried with anhydrous magnesium sulfate and the solvent was removed with a rotary evaporator. Addition of diethyl ether precipitated a light brown solid that was filtered off, washed several times with ether and dried under vacuum (5.13 g, 88% yield).

^1^H NMR (400 MHz, CDCl_3_) δ 8.38 – 8.32 (m, 1H), 8.28 – 8.21 (m, 1H), 8.20 – 8.13 (m, 2H), 7.82 (ddd, *J* = 6.3, 3.4, 1.8 Hz, 2H), 7.59 – 7.49 (m, 3H)

^13^C NMR (101 MHz, CDCl_3_) δ 178.52, 178.05, 175.24, 155.35, 141.70, 134.55, 134.18, 133.22, 132.89, 132.53, 132.19, 129.42, 127.99, 127.94, 127.08

^1^H NMR spectrum of 2-phenylnaphtho[2,3-d]thiazole-4,9-dione

^13^C NMR spectrum of 2-phenylnaphtho[2,3-d]thiazole-4,9-dione

Synthesis of 3-bromo-10-hexyl-10*H*-phenothiazine

10-Hexyl-10*H*-phenothiazine (10 g, 35.42 mmol, M = 282.33 g/mol) was suspended in DCM (150 mL) and *N*-bromosuccinimide (NBS) (6.30 g, 35.42 mmol, M = 177.98 g/mol) dissolved in DCM (100 mL) was slowly added at 0°C. The solution was stirred at room temperature overnight. Water was added. The organic phase was washed several times with water, dried over magnesium sulfate and the solvent removed under reduced pressure. The residue was suspended in pentane. A precipitate formed. It was filtered off, washed several times with pentane. The filtrate was concentrated under reduced pressure. The resulting orange oil was purified by column chromatography (SiO_2_) using pentane as the eluent (10.39 g, 81% yield).

^1^H NMR (400 MHz, CDCl_3_) δ 7.25 – 7.20 (m, 2H), 7.19 – 7.08 (m, 2H), 6.96 – 6.89 (m, 1H), 6.88 – 6.82 (m, 1H), 6.73 – 6.65 (m, 1H), 3.78 (q, *J* = 8.0 Hz, 2H), 1.76 (dq, *J* = 12.6, 7.4 Hz, 2H), 1.47 – 1.36 (m, 2H), 1.36 – 1.22 (m, 4H), 0.87 (dt, *J* = 8.0, 4.5 Hz, 3H).

^13^C NMR (101 MHz, CDCl_3_) δ 145.11, 144.63, 129.94, 129.75, 127.64, 127.57, 127.30, 124.27, 122.77, 116.63, 115.64, 114.50, 47.62, 31.56, 26.86, 26.72, 22.72, 14.14.

^1^H NMR spectrum of 3-bromo-10-hexyl-10*H*-phenothiazine

^13^C NMR spectrum of 3-bromo-10-hexyl-10*H*-phenothiazine

Synthesis of 2-(6,6-dimethylbicyclo[3.1.1]hept-2-en-2-yl)naphtho[2,3-d]thiazole-4,9-dione

A mixture of 2-amino-1,4-naphthoquinone (3.5 g, 20 mmol, M = 173.17 g/mol), (1R)-(-)-myrtenal (3.60 g, 3.65 mL, 24 mmol, M = 150.22 g/mol, d = 0.987), S_8_ (1.9 g, 60 mmol, M = 32.07 g/mol) and NaHCO_3_ (1.7 g, 20 mmol, M = 84.01 g/mol) in DMF (50 mL) were stirred at 100°C overnight. The residue was poured into water (50 mL) and extracted with ethyl acetate. The organic phase was washed several times with water. The combined organic layer was dried with anhydrous magnesium sulfate and the solvent was removed with a rotary evaporator. The residue was purified by column chromatography (SiO_2_) using DCM as the eluent. For a higher purity, addition of a minimum of DCM followed by diethyl ether precipitated a light brown solid that was filtered off, washed several times with ether and dried under vacuum (5.63 g, 84% yield).

^1^H NMR (400 MHz, CDCl_3_) δ 8.37 – 8.26 (m, 1H), 8.26 – 8.15 (m, 1H), 7.87 – 7.71 (m, 2H), 6.90 (tt, *J* = 3.5, 1.5 Hz, 1H), 3.20 (td, *J* = 5.6, 1.7 Hz, 1H), 2.65 – 2.47 (m, 3H), 2.24 (dq, *J* = 7.3, 2.6 Hz, 1H), 1.41 (s, 3H), 1.29 (d, *J* = 9.3 Hz, 1H), 0.87 (s, 3H).

^13^C NMR (101 MHz, CDCl_3_) δ 178.51, 178.15, 175.46, 155.03, 142.47, 140.48, 134.34, 134.03, 133.24, 132.85, 132.71, 127.84, 126.91, 44.36, 40.47, 38.43, 32.69, 31.53, 25.98, 21.03.

^1^H NMR spectrum of 2-(6,6-dimethylbicyclo[3.1.1]hept-2-en-2-yl)naphtho[2,3-d]thiazole-4,9-dione

^13^C NMR spectrum of 2-(6,6-dimethylbicyclo[3.1.1]hept-2-en-2-yl)naphtho[2,3-d]thiazole-4,9-dione

Synthesis of 2-(7-bromo-10-hexyl-10*H*-phenothiazin-3-yl)naphtho[2,3*-d*]thiazole-4,9-dione

A mixture of 2-amino-1,4-naphthoquinone (3.5 g, 20 mmol, M = 173.17 g/mol), 7-bromo-10-hexyl-10*H*-phenothiazine-3-carbaldehyde (9.37 g, 24 mmol, M = 390.34 g/mol), S_8_ (1.9 g, 60 mmol, M = 32.07 g/mol) and NaHCO_3_ (1.7 g, 20 mmol, M = 84.01 g/mol) in DMF (50 mL) were stirred at 100°C overnight. The solution was poured into water (50 mL) and extracted with ethyl acetate. The organic phase was washed several times with water. The combined organic layer was dried with anhydrous magnesium sulfate and the solvent was removed with a rotary evaporator. Addition of a minimum of chloroform followed by diethyl ether precipitated a brown solid that was filtered off, washed several times with ether and dried under vacuum (7.60 g, 66% yield).

NB: Extraction using AcOEt as the solvent seems to be easier. The product seems to be more soluble in AcOEt than in chloroform.

^1^H NMR (400 MHz, CDCl_3_) δ 8.36 – 8.30 (m, 1H), 8.22 (dt, *J* = 6.8, 3.1 Hz, 1H), 7.91 (dd, *J* = 8.5, 2.2 Hz, 1H), 7.87 (d, *J* = 2.1 Hz, 1H), 7.83 – 7.75 (m, 2H), 7.25 (dd, *J* = 7.6, 2.3 Hz, 2H), 6.88 (d, *J* = 8.6 Hz, 1H), 6.72 (d, *J* = 9.0 Hz, 1H), 3.88 – 3.82 (m, 2H), 1.84 – 1.74 (m, 2H), 1.48 – 1.39 (m, 2H), 1.35 – 1.27 (m, 4H), 0.88 (t, *J* = 7.0 Hz, 3H).

^13^C NMR (75 MHz, CDCl_3_) δ 178.13, 177.87, 173.73, 155.22, 148.34, 142.75, 140.73, 134.20, 133.92, 133.12, 132.70, 130.22, 129.73, 127.74, 127.55, 126.80, 126.45, 126.25, 125.85, 124.66, 116.84, 115.51, 115.19, 48.01, 31.35, 26.57, 26.47, 22.56, 13.97.

^1^H NMR spectrum of 2-(7-bromo-10-hexyl-10*H*-phenothiazin-3-yl)naphtho[2,3*-d*]thiazole-4,9-dione

^13^C NMR spectrum of 2-(7-bromo-10-hexyl-10*H*-phenothiazin-3-yl)naphtho[2,3*-d*]thiazole-4,9-dione

Synthesis of 2-(7-bromo-10-hexyl-10*H*-phenothiazin-3-yl)naphtho[2,3-*d*]thiazole-4,9-dione

A mixture of 2-amino-1,4-naphthoquinone (3.5 g, 20 mmol, M = 173.17 g/mol), 10-hexyl-7-nitro-10H-phenothiazine-3-carbaldehyde (8.55 g, 24 mmol, M = 356.44 g/mol), S_8_ (1.9 g, 60 mmol, M = 32.07 g/mol) and NaHCO_3_ (1.7 g, 20 mmol, M = 84.01 g/mol) in DMF (50 mL) were stirred at 100°C overnight. The solution was poured into water (50 mL) and extracted with ethyl acetate. The organic phase was washed several times with water. The combined organic layer was dried with anhydrous magnesium sulfate and the solvent was removed with a rotary evaporator. Addition of a minimum of chloroform followed by diethyl ether precipitated a brown solid that was filtered off, washed several times with ether and dried under vacuum (8.34 g, 77% yield).

^1^H NMR (300 MHz, CDCl_3_) δ 8.66 (d, *J* = 2.2 Hz, 1H), 8.37 – 8.30 (m, 2H), 8.26 – 8.20 (m, 1H), 7.81 (dd, *J* = 6.2, 2.7 Hz, 2H), 7.39 (d, *J* = 9.1 Hz, 1H), 7.29 (d, *J* = 8.8 Hz, 2H), 7.02 (dd, *J* = 8.9, 2.7 Hz, 1H), 4.22 – 4.13 (m, 2H), 1.94 (s, 2H), 1.54 (s, 2H), 1.43 – 1.36 (m, 4H), 0.94 (t, *J* = 7.0 Hz, 3H).

^13^C NMR (75 MHz, CDCl_3_) δ 178.07, 177.87, 173.16, 155.25, 142.64, 140.86, 140.74, 134.27, 134.00, 133.10, 132.67, 131.96, 131.46, 129.58, 127.75, 126.84, 124.87, 124.30, 123.07, 120.96, 117.33, 116.00, 115.79, 48.38, 31.40, 26.45, 22.63, 14.00.

^1^H NMR spectrum of 2-(7-bromo-10-hexyl-10*H*-phenothiazin-3-yl)naphtho[2,3-*d*]thiazole-4,9-dione

^13^C NMR spectrum of 2-(7-bromo-10-hexyl-10*H*-phenothiazin-3-yl)naphtho[2,3-*d*]thiazole-4,9-dione
